# Histone Variant HTZ1 Shows Extensive Epistasis with, but Does Not Increase Robustness to, New Mutations

**DOI:** 10.1371/journal.pgen.1003733

**Published:** 2013-08-22

**Authors:** Joshua B. Richardson, Locke D. Uppendahl, Maria K. Traficante, Sasha F. Levy, Mark L. Siegal

**Affiliations:** 1 Center for Genomics and Systems Biology, Department of Biology, New York University, New York, New York, United States of America; 2 Department of Genetics, Stanford University, Stanford, California, United States of America; Stanford University, United States of America

## Abstract

Biological systems produce phenotypes that appear to be robust to perturbation by mutations and environmental variation. Prior studies identified genes that, when impaired, reveal previously cryptic genetic variation. This result is typically interpreted as evidence that the disrupted gene normally increases robustness to mutations, as such robustness would allow cryptic variants to accumulate. However, revelation of cryptic genetic variation is not necessarily evidence that a mutationally robust state has been made less robust. Demonstrating a difference in robustness requires comparing the ability of each state (with the gene perturbed or intact) to suppress the effects of new mutations. Previous studies used strains in which the existing genetic variation had been filtered by selection. Here, we use mutation accumulation (MA) lines that have experienced minimal selection, to test the ability of histone H2A.Z (HTZ1) to increase robustness to mutations in the yeast *Saccharomyces cerevisiae*. HTZ1, a regulator of chromatin structure and gene expression, represents a class of genes implicated in mutational robustness. It had previously been shown to increase robustness of yeast cell morphology to fluctuations in the external or internal microenvironment. We measured morphological variation within and among 79 MA lines with and without HTZ1. Analysis of within-line variation confirms that HTZ1 increases microenvironmental robustness. Analysis of between-line variation shows the morphological effects of eliminating HTZ1 to be highly dependent on the line, which implies that HTZ1 interacts with mutations that have accumulated in the lines. However, lines without HTZ1 are, as a group, not more phenotypically diverse than lines with HTZ1 present. The presence of HTZ1, therefore, does not confer greater robustness to mutations than its absence. Our results provide experimental evidence that revelation of cryptic genetic variation cannot be assumed to be caused by loss of robustness, and therefore force reevaluation of prior claims based on that assumption.

## Author Summary

Natural populations typically harbor much genetic variation. Some of this variation is cryptic — it does not affect observed traits except if the organism is exposed to a major environmental or genetic perturbation. One often-proposed explanation for the revelation of cryptic genetic variation is that the perturbation has made the organisms less robust to mutations, thereby revealing effects of previously neutral mutations that natural selection had allowed to accumulate. Such effects would be dependent on genetic background, as particular cryptic variants will be present in some individuals and not others. We show that a perturbation of chromatin architecture caused by mutation of the budding-yeast gene HTZ1, encoding histone H2A.Z, does alter phenotypes in a genetic background-dependent manner but does not reduce mutational robustness. Using a large set of yeast lines that accumulated mutations with minimal natural selection, we compared variation in cell morphology with and without HTZ1. The effect of eliminating HTZ1 was highly line dependent, suggesting that HTZ1 interacts extensively with genetic variation in the lines. However, HTZ1+ lines span a range of phenotypes similar to that of the corresponding HTZ1− lines. Our results therefore call into question prior studies linking revelation of cryptic genetic variation with reduced mutational robustness.

## Introduction

Biological systems produce phenotypes that appear to be robust to genetic and non-genetic sources of variation [1], [2]. It has been proposed that understanding robustness is crucial for understanding healthy and diseased states [3]–[5], as well as the potential for populations to adapt to evolutionary pressures [6], [7]. Advancing this understanding will require much greater knowledge of the mechanisms by which robustness is achieved [2].

A particularly important gap in our understanding is that no specific gene product has been shown to confer robustness against naturally occurring mutations, over and above some baseline level of robustness that would exist in the absence of the gene product [8]. It might come as a surprise to some people that this lacuna exists. After all, there is a long history of studies showing that various perturbations, including loss or impairment of specific gene products, reveal previously hidden (“cryptic”) genetic variation [2], [8]–[16]. The earliest study was by Waddington, who observed a crossveinless wing phenotype in *Drosophila melanogaster* only after heat stress and only in some individuals [11]. The basis of the crossveinless phenotype was genetic, as it could be selected for, and lines were established in which the wing phenotype was highly penetrant even without exposure to heat stress [11]. The most prominent recent example of such an experiment involves the molecular chaperone Hsp90, the impairment of which reveals cryptic variation in several evolutionarily distant species [10]. In flies, impairment of HSP90 reveals phenotypic variation in several traits, and, as in the Waddington experiments, this variation is heritable [12]. Although recent work has shown that severe Hsp90 impairment induces mutations via mobilization of transposable elements [17], new mutations do not explain all revealed variation [18], [19]. Other recent work has expanded the scope of studies of cryptic genetic variation to other model organisms, including the nematode worm *Caenorhabditis elegans* [20], [21], the budding yeast *Saccharomyces cerevisiae* [22]–[24] and the bacterium *Escherichia coli* [25]. Such studies have also expanded to non-model species, including tobacco hornworms [26], dung flies [27] and a beetle-associated nematode [28]. The list of genes in *D. melanogaster* whose impairment can reveal cryptic variation is poised to expand as well: a genetic screen using deficiency chromosomes recently showed that there are at least 10 regions of the *D. melanogaster* genome containing a gene that reveals cryptic variation in wing morphology when hemizygous [29].

**Figure 1:**
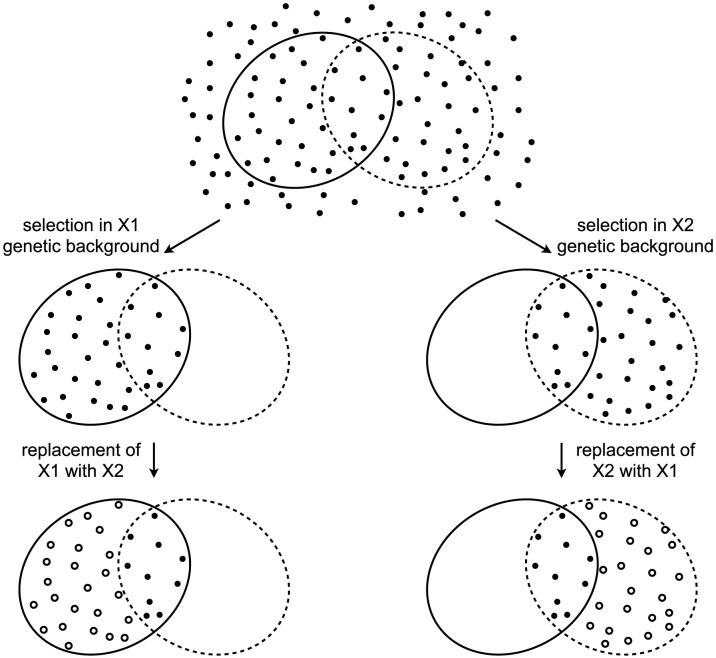
Revelation of cryptic genetic variation without a change in robustness. Top: In an abstract space of possible mutations (points), some (surrounded by solid ellipse) are neutral in the context of an allele X1 and some (surrounded by dashed ellipse) are neutral in the context of an allele X2. Alleles X1 and X2 confer equal robustness to mutations because an equal number of mutations are neutral in the context of each. Middle: Under selection, only neutral mutations accumulate. In the X1 genetic background, these are the mutations within the solid ellipse (left), whereas in the X2 genetic background these are the mutations within the dashed ellipse (right). Bottom: Perturbing the system by replacing one X allele with the other reveals cryptic genetic variation (open circles).

The decades of studies revealing cryptic variation have been taken as evidence that the wild-type state is more robust to mutations than the perturbed state, and that the perturbed gene products normally contribute to this robustness. However, this logic is flawed [8]. To appreciate the logical flaw, consider the following example. A gene X has two alleles, X1 and X2, that confer equal robustness against mutations entering a population. That is, the distribution of mutational effects, including the proportion of mutations with no phenotypic effect (neutral mutations), is identical in the X1 and X2 genetic backgrounds. In this example, the only difference between X1 and X2 is in which mutations they make neutral (Figure 1). A population fixed for X1 will accumulate the mutations that are neutral in the context of X1. Replacing X1 with X2 in members of the population will reveal cryptic genetic variation — the subset of mutations that are neutral in the X1 genetic background but non-neutral in the X2 genetic background. Likewise, a population fixed for X2 will accumulate the mutations that are neutral in the context of X2, a subset of which are non-neutral in the context of X1. Replacing X2 with X1 will therefore also reveal cryptic genetic variation. It is thus clear that the revelation of cryptic genetic variation is not sufficient to indicate a decrease in robustness. Indeed, cryptic genetic variation can be revealed even by a perturbation that makes the system more, not less, robust. The revelation of cryptic genetic variation by a perturbation merely indicates that some mutations are conditionally neutral; it does not indicate whether the perturbation is more likely to convert a neutral allele into one with a phenotypic effect than to do the reverse [8].

To ascertain the relative amount of mutational robustness conferred by alternative alleles, it is necessary to measure, in the context of each allele, the phenotypic effects of a large sample of spontaneous mutations. As has been noted [8], an approximation of this experiment has been conducted in *D. melanogaster* [30] and *E. coli* [31], [32]. However, the mutations assayed in those studies had survived the filter of natural selection, and so a major assumption had to be made that the lines had not experienced selection toward a common optimum [8]. The most appropriate sample of mutations must include those mutations that would otherwise be purged by selection in the presence of one or the other allele [8]. One way to construct the appropriate sample is to allow mutations to accumulate in independent lines by serial passaging through bottlenecks. The bottlenecks keep the effective population size low and therefore minimize the power of natural selection to purge deleterious alleles. Then a corresponding set of lines in which one allele is replaced with the other allele can be constructed. The difference in phenotypic variation between the two sets of lines — or, in the parlance of quantitative genetics, the difference in their mutational variances [33]–[35] — indicates the relative robustness conferred by the two alleles.

Here, we perform this test using strains of *S. cerevisiae*. Yeast, due to their rapid growth rate, ease of handling, and routine genetic techniques, are highly amenable to large-scale experiments such as this one. To avoid the problem of using organisms maintained under selection when testing the extent of mutational robustness conferred by a candidate gene, we use yeast lines generated in a mutation accumulation (MA) experiment [36], [37]. In this MA experiment, 151 replicate lines were founded from a single diploid ancestral strain, and cultured independently for approximately 2062 generations with single-individual bottlenecks at approximately 20-generation intervals [37]. The use of a diploid ancestor promoted genome stability [38] and, because propagation was asexual, completely shielded recessive deleterious mutations from selection. Based on estimates from a different MA experiment [39], each line should contain approximately 8 single-nucleotide changes (point mutations) per haploid genome. These strains also likely acquired mutations in repetitive sequences. Again based on prior estimates [39], the expected number of microsatellite mutations per haploid genome per line is approximately 4 and the expected number of mutations in short homopolymer runs per haploid genome per line is approximately 638.

We chose to test if the presence of a particular chromatin regulator, HTZ1, confers greater robustness to new mutations than its absence. HTZ1 encodes a histone variant, H2A.Z, that can take the place of histone H2A in nucleosomes. Nucleosomes containing HTZ1 are usually found in the promoter regions of repressed or stress-responsive genes, and HTZ1 is necessary for these genes' full activation [40]. Despite its widespread effects on gene regulation, HTZ1 is not required for viability and indeed its deletion has only a modest effect on growth, making it a convenient choice for genetic analysis of robustness. Moreover, HTZ1 was identified in a systematic screen for yeast genes that contribute to robustness to microenvironmental sources of variation, including the immediate external environment as well as internal stochastic processes [41]. That screen identified a few hundred genes that, when absent, significantly increased variance of many cell-shape traits [41]. Genes involved in chromosome organization were over-represented among the significant genes, and HTZ1 was one of the most highly significant [41]. Although only microenvironmental sources of variation were present in that study, as cells were genetically identical and cultured together, several lines of argument have led many to predict that genes contributing to robustness to one source of variation will increase robustness to other sources as well [2], [42], [43].

The prediction is especially strong for chromatin regulators, which have been found in other studies to affect levels of phenotypic variation due to genetic and systematic environmental differences [44]–[46]. A recent study found that chromatin regulators suppress gene expression differences between *S. cerevisiae* and its close relative *S. paradoxus* [44]. Orthologous genes show similar expression profiles in wild-type strains of each species. However, the expression profiles became more dissimilar when any one of eight chromatin regulators, including HTZ1, was deleted [44]. A study that investigated chemical-protein relationships in *S. cerevisiae* found that although chromatin regulators did not directly interact with many chemicals, their presence increased resistance to many chemicals [45]. In Drosophila, impairment of a chromatin-regulator network causes developing flies to be more sensitive to temperature variation [46].

We knocked out HTZ1 in 79 MA lines. We first converted the existing diploid MA lines [37] to haploids to study the effects of accumulated mutations without the complication of dominance. We did this conversion before deleting HTZ1 in each line so that the same sample of mutations would be assayed in the presence of HTZ1 as in the absence of HTZ1. That is, we created 79 strain pairs, with the members of each pair having identical genotype except at the HTZ1 locus.

We measured phenotypic variation in cell morphology in each strain, using an established assay [47] that we adapted for higher throughput. In this assay, cells are fixed and stained with fluorescent markers of the cell surface and the nucleus, then imaged. Based on these markers, CalMorph image-analysis software automatically measures 187 cell shape parameters, such as cell diameter and budding angle [47]. We present an analysis of phenotypic variation within and among HTZ1+ and HTZ1− lines as a test of the contribution of wild-type HTZ1 function to robustness.

## Results

### Elimination of HTZ1 increases within-line variation

The main goal of this study was to test whether the chromatin protein HTZ1 increases robustness of morphological phenotypes to new mutations, by collecting morphological data on 79 pairs of HTZ1+ and HTZ1− MA lines. As described in Materials and Methods, morphological measurements of individual cells were obtained by adapting an established method of automated image analysis of fluorescence micrographs [47]. HTZ1 was chosen as a candidate mutational-robustness factor in part because it had previously been found to confer robustness to microenvironmental variation, in that morphological variation increased among genetically identical cells when HTZ1 was deleted [41]. Before addressing our main goal, we therefore first sought to confirm this previous finding, by asking if HTZ1 deletion increases within-line variation of morphological phenotypes.

Because the morphological assay consists of partially redundant phenotypes, we used principal component analysis (PCA) to identify orthogonal linear combinations of the phenotypes to use for downstream quantifications of morphological variation. PCA was performed separately for the three cell types (unbudded, small-budded and large-budded), because each type has its own suite of phenotype measurements. Only principal components that explained more variance than the random expectation were used in the analysis (see Materials and Methods). This reduced the dimensionality of the data to six significant principal components for unbudded cells, 10 for small-budded cells and 17 for large-budded cells (Figure S1).

We estimated variance parameters by fitting a linear model. A standard approach to doing so would be to use maximum-likelihood based methods to fit mixed models in which genotype is a fixed effect and MA line is a random effect. However, we chose instead to estimate variance components using a Bayesian approach based on Markov chain Monte Carlo (MCMC) sampling (see Materials and Methods). The MCMC approach has the advantages of: 1) high flexibility in modeling different within-line and between-line variances for each HTZ1 genotype, and 2) straightforward assessment of the precision of variance estimates by constructing credible intervals from the posterior distributions of the parameters.

We compared estimates of within-line variance for HTZ1+ and HTZ1− lines to determine the effect of HTZ1 on microenvironmental robustness. As shown in Table 1, for each of the 33 principal components, the within-line variance is greater in HTZ1− lines than in HTZ1+ lines. The differences are substantial: in only three cases is there overlap of the estimates' 95% highest posterior density (HPD) intervals (credible intervals that are akin to confidence intervals but computed as the shortest intervals containing 95% of the posterior-distribution density). Likewise, in only these three cases did the 95% credible interval for the difference between the HTZ1− and HTZ1+ within-line variances include 0 or negative values. To confirm that this result was not due to any unknown bias of the MCMC approach, we also compared within-line variances using a model-independent approach. We compared median-corrected median absolute deviations (a robust measure of within-line spread) between HTZ1+ and HTZ1− lines and found, as expected, lower within-line spread for HTZ1+ than for HTZ1− (see Text S1, Figure S2). These results confirm that HTZ1 mutation increases within-line variation, as shown in our previous study [41]. That is, the results confirm that HTZ1+ increases robustness to microenvironmental sources of variation, and suggest this ability is not dependent on the line background.

**Table 1:** Estimates of within-line variance, along with 95% credible intervals (CrI), for HTZ1+ lines and HTZ1− lines, derived from MCMC.

Cell Type	PC		HTZ1+	CrI lower bound HTZ1+	CrI upper bound HTZ1+	HTZ1−	CrI lower bound HTZ1−	CrI upper bound HTZ1−
No Bud	1	*	0.722	0.716	0.731	1.116	1.103	1.128
No Bud	2	*	0.713	0.706	0.720	1.177	1.164	1.191
No Bud	3	*	0.702	0.697	0.711	0.830	0.820	0.839
No Bud	4	*	0.891	0.881	0.899	0.954	0.946	0.967
No Bud	5	*	0.745	0.737	0.752	1.059	1.047	1.071
No Bud	6	*	0.867	0.858	0.874	1.116	1.104	1.128
Small Bud	1	*	0.805	0.795	0.816	1.098	1.083	1.114
Small Bud	2	*	0.825	0.812	0.834	1.074	1.063	1.092
Small Bud	3	*	0.776	0.764	0.785	1.088	1.074	1.105
Small Bud	4	*	0.860	0.847	0.870	1.108	1.091	1.123
Small Bud	5	*	0.715	0.703	0.722	0.838	0.828	0.852
Small Bud	6	*	0.918	0.907	0.932	1.025	1.012	1.040
Small Bud	7	*	0.922	0.913	0.938	1.052	1.039	1.070
Small Bud	8	*	0.926	0.915	0.940	1.029	1.017	1.046
Small Bud	9	*	0.873	0.862	0.885	1.001	0.989	1.017
Small Bud	10	*	0.955	0.940	0.966	1.017	1.006	1.036
Large Bud	1	*	0.748	0.735	0.760	0.995	0.980	1.020
Large Bud	2		0.987	0.972	1.008	1.013	0.991	1.033
Large Bud	3		0.973	0.955	0.988	0.991	0.973	1.015
Large Bud	4	*	0.766	0.754	0.780	0.973	0.956	0.993
Large Bud	5	*	0.924	0.911	0.942	1.039	1.017	1.059
Large Bud	6	*	0.872	0.860	0.889	1.082	1.060	1.105
Large Bud	7	*	0.921	0.905	0.937	1.079	1.054	1.097
Large Bud	8	*	0.785	0.769	0.794	1.015	0.993	1.035
Large Bud	9	*	0.750	0.736	0.761	1.083	1.065	1.109
Large Bud	10	*	0.654	0.641	0.663	0.756	0.745	0.776
Large Bud	11	*	0.912	0.893	0.925	1.014	1.002	1.042
Large Bud	12	*	0.908	0.892	0.923	1.028	1.010	1.051
Large Bud	13	*	0.859	0.845	0.874	1.117	1.094	1.139
Large Bud	14	*	0.924	0.908	0.939	1.051	1.025	1.064
Large Bud	15	*	0.976	0.958	0.991	1.015	0.991	1.032
Large Bud	16		0.980	0.966	0.999	1.006	0.984	1.022
Large Bud	17	*	0.889	0.875	0.905	1.127	1.111	1.156

* = principal component (PC) for which the CrI of the difference between HTZ1+ and HTZ1− in the within-line variance estimates does not overlap 0.

### HTZ1+ and HTZ1− lines exhibit similar ranges of morphological variation

**Figure 2:**
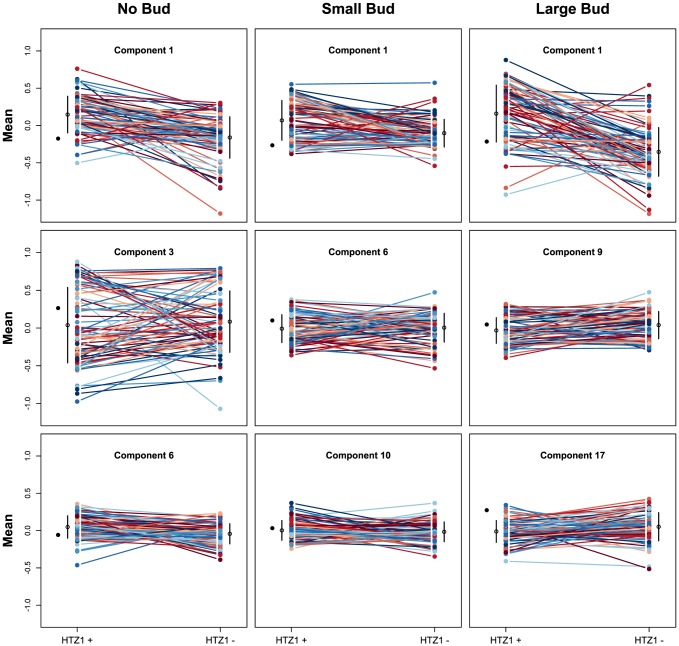
Mean principal component values of HTZ1+ and HTZ1− lines. Each line connects an HTZ1+ MA line with its HTZ1− derivative. The means and standard deviations of line means are indicated by the black circles and bars. The mean of the ancestral strain is shown to the left in each plot.

We next asked if HTZ1 affects between-line morphological variation and consequently affects robustness to new mutations. Figure 2 shows the HTZ1+ and HTZ1− line means for three principal components from each cell type (see Figure S3 for the remaining principal components from each cell class). MCMC-based estimates of the between-line variances of HTZ1+ and HTZ1− lines and 95% credible intervals are shown in Table 2. For nine of 33 principal components, the 95% credible intervals for the difference between the HTZ1+ and HTZ1− between-line variances do not include 0. In four of these cases, the HTZ1− lines have higher between-line variance, whereas in the other five the HTZ1+ lines have higher between-line variance. As above for the within-line variance comparison, we used a model-independent test to corroborate the MCMC-based analysis. Using Levene's test for differences in between-line variance yielded qualitatively similar results (see Text S1, Table S1). Taken together, these results demonstrate that HTZ1 does not systematically affect between-line variance, especially considering that the principal components that showed a significant difference did not consistently show an effect in the same direction.

The absence of a systematic effect on between-line variance supports the scenario diagrammed in Figure 1, where neither HTZ1+ nor HTZ1− contributes more to genetic robustness than the other. Instead, each HTZ1 allele interacts epistatically with a certain subset of accumulated mutations to produce the range of morphological variation seen in this experiment. The subsets for the two alleles may overlap only partially, but their sizes, as measured by the alleles' effects on morphology, are similar.

**Figure 3:**
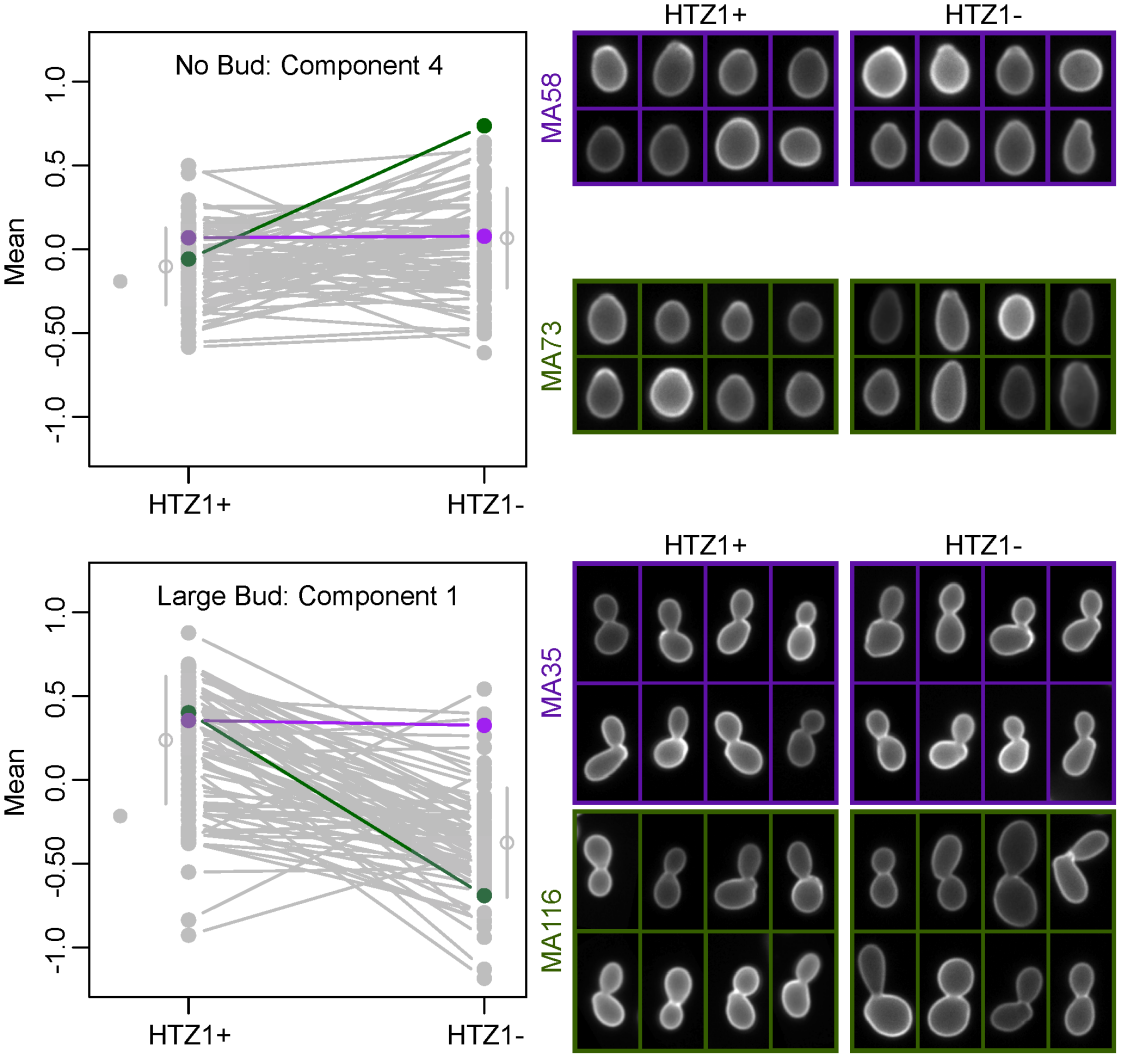
Differences in principal component values reflect underlying differences in morphological phenotypes. Individual cells from line pairs with similar (purple) and dissimilar (green) mean principal component values are shown for two different principal components. The line plots to the left are identical to those in Figure 2 or Figure S3, but with all lines grayed out except for those corresponding to the MA lines depicted on the right.

Because principal components represent combinations of morphological trait values, as opposed to an individual trait such as cell circumference or budding angle, relating a principal component to a biologically meaningful phenotype can be difficult. However, inspection of individual cells from line pairs with divergent mean principal component values indicates that this difference accurately reflects underlying differences in cell morphology. For example, consistent with which original phenotypes load heavily onto each principal component, principal component 4 for unbudded cells appears to correspond to how elongated a cell is, whereas principal component 1 for large-budded cells appears to correspond to cell size (Figure 3).

### HTZ1 has a strain-dependent effect on morphology

In principle, the absence of consistent, major differences in between-line variance could be caused either by the lack of any effect of HTZ1 genotype on line means or by a significant genotype-by-line interaction effect that takes the form of line crossing rather than line spreading. Note that “line” here refers not to MA line, but to a line as a geometric object connecting the means of the two HTZ1 genotypes of the same MA line in a plot such as Figure 2. That is, line crossing refers to the change in rank order of line means, and line spreading refers to the change in dispersion of line means. Figure 2 and Figure S3 appear to indicate a large extent of line crossing. To measure variance due to genotype-by-line interaction, and to partition this interaction into components representing line crossing and line spreading, we used the variance components estimated by MCMC. The genotype-by-line interaction variance, V_g × l_, is estimated as [48], [49]:
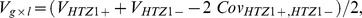
where V_HTZ1+_ is the HTZ1+ between-line variance, V_HTZ1−_ is the HTZ1− between-line variance and Cov_HTZ1+,HTZ1−_ is the genetic covariance between HTZ1+ and HTZ1−. A test of the significance of the genotype-by-line interaction is not possible with the MCMC approach, because: 1) the use of an information criterion that penalizes additional parameters, akin to the Akaike information criterion or Bayesian information criterion, is not well established for model selection in this context; and 2) variances are constrained to be positive and therefore credible intervals will not overlap 0 even for negligible variances. Nevertheless, an indication that the interaction variance is substantial is that it is similar in magnitude to the magnitudes of the between-line variances (compare Table 2 to Table 3, which shows the interaction-variance estimates and their 95% credible intervals). An equivalent way of saying this is that the genetic correlation is far from unity [48], as is indeed the case for the correlation between HTZ1+ and HTZ1− lines for each principal component. It is possible to perform a significance test for nested models fit by maximum-likelihood approaches. We did this, applying a likelihood-ratio test to models with and without an interaction term, and found that models containing the interaction term fit the data better than models without, for all principal components (see Text S1, Table S1).

**Table 2:** Estimates of between-line variance, along with 95% credible intervals (CrI), for HTZ1+ lines and HTZ1− lines, derived from MCMC.

Cell Type	PC		HTZ1+	CrI lower bound HTZ1+	CrI upper bound HTZ1+	HTZ1−	CrI lower bound HTZ1−	CrI upper bound HTZ1−
No Bud	1		0.0806	0.0552	0.1059	0.0612	0.0448	0.0831
No Bud	2		0.0498	0.0377	0.0730	0.0554	0.0410	0.0774
No Bud	3	†	0.1561	0.1184	0.2213	0.2293	0.1805	0.3446
No Bud	4	*	0.0872	0.0659	0.1237	0.0523	0.0359	0.0709
No Bud	5		0.1081	0.0785	0.1529	0.0846	0.0613	0.1171
No Bud	6		0.0167	0.0118	0.0235	0.0213	0.0152	0.0301
Small Bud	1	†	0.0343	0.0234	0.0454	0.0699	0.0512	0.0975
Small Bud	2		0.0476	0.0332	0.0646	0.0313	0.0218	0.0421
Small Bud	3	*	0.0699	0.0515	0.0990	0.0456	0.0302	0.0601
Small Bud	4	*	0.0426	0.0293	0.0584	0.0115	0.0081	0.0170
Small Bud	5		0.1851	0.1246	0.2349	0.2548	0.1780	0.3291
Small Bud	6		0.0305	0.0242	0.0484	0.0324	0.0235	0.0452
Small Bud	7		0.0152	0.0109	0.0229	0.0167	0.0117	0.0237
Small Bud	8		0.0196	0.0150	0.0306	0.0157	0.0128	0.0253
Small Bud	9	†	0.0416	0.0297	0.0569	0.0832	0.0647	0.1189
Small Bud	10		0.0156	0.0108	0.0219	0.0163	0.0111	0.0223
Large Bud	1		0.1020	0.0720	0.1440	0.1442	0.1043	0.1943
Large Bud	2		0.0219	0.0146	0.0309	0.0312	0.0227	0.0462
Large Bud	3	†	0.1135	0.0844	0.1585	0.2155	0.1574	0.3004
Large Bud	4	*	0.0721	0.0496	0.0977	0.0339	0.0236	0.0471
Large Bud	5		0.0826	0.0580	0.1170	0.0917	0.0647	0.1243
Large Bud	6		0.0305	0.0202	0.0421	0.0214	0.0150	0.0321
Large Bud	7		0.0377	0.0292	0.0594	0.0450	0.0308	0.0610
Large Bud	8		0.0249	0.0189	0.0392	0.0247	0.0168	0.0363
Large Bud	9		0.0298	0.0199	0.0408	0.0259	0.0178	0.0369
Large Bud	10		0.0195	0.0138	0.0313	0.0157	0.0115	0.0252
Large Bud	11		0.0845	0.0602	0.1184	0.0541	0.0389	0.0754
Large Bud	12		0.0082	0.0054	0.0143	0.0063	0.0037	0.0097
Large Bud	13		0.0469	0.0326	0.0657	0.0332	0.0218	0.0441
Large Bud	14		0.0173	0.0115	0.0255	0.0149	0.0103	0.0221
Large Bud	15		0.0176	0.0120	0.0273	0.0219	0.0147	0.0316
Large Bud	16		0.0076	0.0039	0.0113	0.0076	0.0049	0.0123
Large Bud	17	*	0.0303	0.0229	0.0445	0.0178	0.0119	0.0250

* = principal component (PC) for which the CrI of the difference between HTZ1+ and HTZ1− in the between-line variance estimates does not overlap 0, and for which the HTZ1+ between-line variance estimate is higher than that of HTZ1−.

† = PC for which the CrI of the difference between HTZ1+ and HTZ1− in the between-line variance estimates does not overlap 0, and for which the HTZ1+ between-line variance estimate is lower than that of HTZ1−.

**Table 3:** Estimates of genotype-by-line interaction variances and 95% credible intervals (CrI) for each principal component (PC), derived from MCMC.

Cell Type	PC	Interaction variance	CrI lower bound	CrI upper bound
No Bud	1	0.0566	0.0447	0.0854
No Bud	2	0.0446	0.0346	0.0664
No Bud	3	0.1462	0.1046	0.1944
No Bud	4	0.0653	0.0464	0.0904
No Bud	5	0.0832	0.0654	0.1224
No Bud	6	0.0204	0.0148	0.0290
Small Bud	1	0.0415	0.0286	0.0569
Small Bud	2	0.0347	0.0259	0.0507
Small Bud	3	0.0453	0.0318	0.0616
Small Bud	4	0.0315	0.0209	0.0422
Small Bud	5	0.1426	0.1051	0.1986
Small Bud	6	0.0248	0.0176	0.0360
Small Bud	7	0.0178	0.0117	0.0245
Small Bud	8	0.0225	0.0171	0.0333
Small Bud	9	0.0514	0.0380	0.0743
Small Bud	10	0.0146	0.0117	0.0239
Large Bud	1	0.1068	0.0806	0.1526
Large Bud	2	0.0161	0.0108	0.0243
Large Bud	3	0.1189	0.0949	0.1808
Large Bud	4	0.0401	0.0302	0.0589
Large Bud	5	0.0771	0.0579	0.1115
Large Bud	6	0.0267	0.0182	0.0379
Large Bud	7	0.0347	0.0266	0.0522
Large Bud	8	0.0151	0.0121	0.0262
Large Bud	9	0.0187	0.0119	0.0262
Large Bud	10	0.0150	0.0108	0.0241
Large Bud	11	0.0590	0.0451	0.0886
Large Bud	12	0.0071	0.0037	0.0103
Large Bud	13	0.0381	0.0270	0.0540
Large Bud	14	0.0141	0.0095	0.0211
Large Bud	15	0.0136	0.0090	0.0210
Large Bud	16	0.0064	0.0034	0.0105
Large Bud	17	0.0241	0.0153	0.0318

A genotype-by-line interaction term can be partitioned into terms representing the spreading of line means and the crossing of line means [30], [48], [49], as described in Materials and Methods. The percentages of the interaction variance explained by line crossing and spreading, along with credible intervals, are reported in Table 4. For each principal component, the vast majority of the genotype-by-line interaction is indeed explained by line crossing, rather than the spreading of line means (median percentage of interaction explained by line crossing = 99.9%; Table 4, column 3). We obtained similar results when using variance estimates from models fit by restricted maximum likelihood (see Text S1, Figure S4, Table S1). Note that, for the principal component with the highest estimate of the spreading component (small-budded principal component 4, estimated percentage spreading = 12.46%), the HTZ1+ between-line variance is higher than the HTZ1− between-line variance. That is, the spreading is in the direction of the wild type rather than the mutant.

**Table 4:** Estimates of percentage of interaction variance explained by crossing of line means or spreading of line means, along with 95% credible intervals (CrI), for each principal component (PC), derived from MCMC.

Cell Type	PC	Crossing	CrI lower bound Crossing	CrI upper bound Crossing	Spreading	CrI lower bound Spreading	CrI upper bound Spreading
No Bud	1	99.97	94.67	100.00	0.03	1.19×10^−6^	5.33
No Bud	2	99.97	97.10	100.00	0.03	2.84×10^−7^	2.90
No Bud	3	99.92	89.32	100.00	0.08	1.05×10^−4^	10.68
No Bud	4	97.74	89.10	100.00	2.26	3.33×10^−4^	10.90
No Bud	5	99.97	93.65	100.00	0.03	1.30×10^−6^	6.35
No Bud	6	99.98	95.47	100.00	0.02	6.32×10^−7^	4.53
Small Bud	1	92.13	80.70	99.24	7.87	7.65×10^−1^	19.30
Small Bud	2	99.93	91.72	100.00	0.07	5.82×10^−6^	8.28
Small Bud	3	99.94	88.37	100.00	0.06	2.93×10^−6^	11.63
Small Bud	4	87.54	74.15	95.24	12.46	4.76×10^0^	25.85
Small Bud	5	99.95	91.97	100.00	0.05	3.86×10^−5^	8.03
Small Bud	6	99.98	96.05	100.00	0.02	8.47×10^−8^	3.95
Small Bud	7	99.98	96.90	100.00	0.02	2.05×10^−6^	3.10
Small Bud	8	99.98	96.27	100.00	0.02	2.63×10^−8^	3.73
Small Bud	9	93.49	82.48	99.13	6.51	8.65×10^−1^	17.52
Small Bud	10	99.97	97.08	100.00	0.03	2.27×10^−8^	2.92
Large Bud	1	99.95	92.95	100.00	0.05	1.56×10^−6^	7.05
Large Bud	2	99.93	87.67	100.00	0.07	4.19×10^−5^	12.33
Large Bud	3	92.96	85.69	100.00	7.04	3.17×10^−6^	14.31
Large Bud	4	93.76	82.51	99.99	6.24	7.44×10^−3^	17.49
Large Bud	5	99.99	96.70	100.00	0.01	3.45×10^−7^	3.30
Large Bud	6	99.94	93.66	100.00	0.06	2.26×10^−6^	6.34
Large Bud	7	99.97	96.78	100.00	0.03	1.34×10^−8^	3.22
Large Bud	8	99.97	95.29	100.00	0.03	3.60×10^−6^	4.71
Large Bud	9	99.97	95.04	100.00	0.03	4.76×10^−7^	4.96
Large Bud	10	99.98	93.19	100.00	0.02	3.95×10^−6^	6.81
Large Bud	11	99.95	90.05	100.00	0.05	2.65×10^−6^	9.95
Large Bud	12	99.90	85.20	100.00	0.10	5.05×10^−5^	14.80
Large Bud	13	99.94	92.52	100.00	0.06	2.70×10^−6^	7.48
Large Bud	14	99.98	94.66	100.00	0.02	2.71×10^−7^	5.34
Large Bud	15	99.95	92.93	100.00	0.05	1.99×10^−8^	7.07
Large Bud	16	99.95	92.98	100.00	0.05	4.31×10^−7^	7.02
Large Bud	17	97.55	86.31	100.00	2.45	5.91×10^−6^	13.69

To confirm that our conclusions were not dependent on the method of dimensional reduction, we repeated our interaction analysis using partitioning around medoids (PAM) instead of PCA, as we had done previously to reduce phenotypic redundancies [41]. The number of clusters used in a PAM analysis is often chosen to maximize average silhouette width [50]. Alternatively, the number of significant principal components (determined as described above) can be taken as an appropriate number of clusters. For our data, the number of significant principal components is smaller than the number of clusters with the highest mean silhouette width. However, the mean silhouette width corresponding to this smaller number of clusters is very similar to the maximum mean silhouette width, suggesting that adding more clusters than the number corresponding to the number of significant principal components does not improve the clustering much (Figure S5). We therefore used the smaller number of clusters. In all respects, our main findings were not altered when repeating analysis with PAM instead of PCA. HTZ1− lines have greater within-line variance for each medoid, and the differences are substantial (Table S2). In only two cases did the 95% credible interval of the difference between within-line variances overlap 0. Using medoids instead of principal components resulted in 11 of 33 medoids showing evidence of a difference in between-line variance, in the form of the 95% credible interval of the difference in between-line variance not overlapping 0. For six of these 11, the HTZ1+ lines showed higher between-line variance, and for the other five the HTZ1− lines showed higher between-line variance (Table S3). The magnitude of the genotype-by-line interaction variance was similar to the magnitude of the between-line variance, as in the PCA-based analysis. In addition, the model-selection analysis of models fit by maximum likelihood showed that for 32 of 33 medoids the best-fit model includes an interaction term (Text S1, Table S4).

### Sensitivity of results to number and identity of MA lines tested

As noted in Materials and Methods, the MA lines and their HTZ1− derivatives are expected to produce red colonies due to the presence of an *ade2-101* mutation, yet a subset of lines displayed white sectors or colonies, suggesting an epigenetic switch was at play. We therefore repeated analyses with the restricted set of line pairs that showed stable-red inheritance. The restricted analysis yields the same general conclusions as the analysis with the full set of strains. For each principal component, HTZ1− lines have a greater within-line variance. For only two principal components did the 95% credible interval of the difference between within-line variances overlap 0. In addition, for only four principal components did the credible intervals of the difference in between-line variances not overlap 0. For three of these four, the HTZ1− lines had greater between-line variance and in one the HTZ1+ lines did. For each principal component, the magnitude of the between-line variance and genotype-by-line interaction variance is similar. The model-selection analysis showed that for all principal components, the best-fit model included an interaction term.

One potential, related concern about our experimental approach is that estimates of line means and between-line variances might be sensitive to the number of lines assayed. However, it is unlikely that sampling any more lines would change our results. Estimates of the mean and standard deviation of line means for various principal components were recalculated with a line sample size ranging from 5 to 79 pairs. As shown in Figure S6 for the example of principal component 1 for unbudded cells, these estimates do not change greatly even in the range where the number of lines is approximately half of the number we used, and they show extremely small differences as the number of lines approaches 79. This observation suggests that sampling more MA lines would not change the results of this experiment, and that the amount of morphological variation caused by accumulated mutations has been adequately measured.

### Estimates of mutational variance

**Figure 4:**
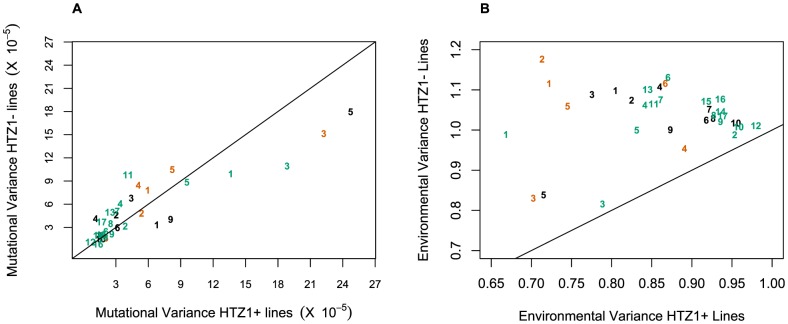
Mutational and environmental variances in HTZ1+ and HTZ1− lines. (A) Mutational variances estimated for the HTZ1+ and HTZ1− lines for the indicated principal component. Orange = principal components for no-bud phenotypes; black = principal components for small-bud phenotypes; green = principal components for large-bud phenotypes. (B) Environmental variances estimated for the HTZ1+ and HTZ1− lines for the indicated principal component. Color scheme is the same as for A.

An alternative (although clearly not independent) way of framing the question of whether HTZ1 increases robustness to mutations is to compare the mutational variance, V_M_, estimated from the HTZ1+ MA lines to that estimated from the HTZ1− MA lines. If HTZ1+ were to contribute to greater robustness to mutations, then the V_M_ should be higher for the HTZ1− lines than the HTZ1+ lines. The magnitude of V_M_ (scaled by the environmental variance, V_E_) is also of interest, as it relates to the mutational target size for the trait of interest and the neutral expectation for segregating variation [33], [34]. V_M_ for cell morphology, which had not been previously estimated, was estimated for lines with and without HTZ1 (see Materials and Methods). The V_M_ estimates and their 95% credible intervals are given in Table 5 for HTZ1− and HTZ1+ lines for each principal component. The average V_M_/V_E_ of HTZ1− principal components is 5.1×10^−5^ and of HTZ1+ principal components is 7.8×10^−5^. Plots of V_M_ and V_E_ estimates for each principal component in HTZ1− versus HTZ1+ lines are shown in Figure 4, and capture our main conclusions: HTZ1 mutation increases environmental variance but does not increase mutational variance.

**Table 5:** Estimates of mutational variance and 95% credible intervals (CrI), derived from MCMC.

Cell Type	PC	HTZ1−	CrI lower bound HTZ1−	CrI upper bound HTZ1−	HTZ1+	CrI lower bound HTZ1+	CrI upper bound HTZ1+
No Bud	1	7.81×10^−5^	5.35×10^−5^	1.03×10^−4^	5.94×10^−5^	4.34×10^−5^	8.06×10^−5^
No Bud	2	4.83×10^−5^	3.66×10^−5^	7.08×10^−5^	5.38×10^−5^	3.98×10^−5^	7.50×10^−5^
No Bud	3	1.51×10^−4^	1.15×10^−4^	2.15×10^−4^	2.22×10^−4^	1.75×10^−4^	3.34×10^−4^
No Bud	4	8.46×10^−5^	6.39×10^−5^	1.20×10^−4^	2.08×10^−5^	3.48×10^−5^	6.88×10^−5^
No Bud	5	1.05×10^−4^	7.62×10^−5^	1.48×10^−4^	8.21×10^−5^	5.95×10^−5^	1.14×10^−4^
No Bud	6	1.62×10^−5^	1.14×10^−5^	2.28×10^−5^	2.07×10^−5^	1.48×10^−5^	2.92×10^−5^
Small Bud	1	3.33×10^−5^	2.27×10^−5^	4.41×10^−5^	6.78×10^−5^	4.96×10^−5^	9.46×10^−5^
Small Bud	2	4.61×10^−5^	3.22×10^−5^	6.26×10^−5^	3.03×10^−5^	2.12×10^−5^	4.08×10^−5^
Small Bud	3	6.78×10^−5^	5.00×10^−5^	9.60×10^−5^	4.42×10^−5^	2.93×10^−5^	5.83×10^−5^
Small Bud	4	4.13×10^−5^	2.84×10^−5^	5.66×10^−5^	1.12×10^−5^	7.84×10^−6^	1.65×10^−5^
Small Bud	5	1.80×10^−4^	1.21×10^−4^	2.28×10^−4^	2.47×10^−4^	1.73×10^−4^	3.19×10^−4^
Small Bud	6	2.96×10^−5^	2.35×10^−5^	4.70×10^−5^	3.14×10^−5^	2.28×10^−5^	4.39×10^−5^
Small Bud	7	1.48×10^−5^	1.06×10^−5^	2.22×10^−5^	1.62×10^−5^	1.13×10^−5^	2.30×10^−5^
Small Bud	8	1.90×10^−5^	1.45×10^−5^	2.97×10^−5^	1.53×10^−5^	1.24×10^−5^	2.45×10^−5^
Small Bud	9	4.04×10^−5^	2.88×10^−5^	5.52×10^−5^	8.07×10^−5^	6.28×10^−5^	1.15×10^−4^
Small Bud	10	1.51×10^−5^	1.05×10^−5^	2.12×10^−5^	1.58×10^−5^	1.08×10^−5^	2.16×10^−5^
Large Bud	1	9.94×10^−5^	5.72×10^−5^	1.45×10^−4^	1.36×10^−5^	9.26×10^−5^	2.37×10^−4^
Large Bud	2	3.16×10^−5^	1.95×10^−5^	5.19×10^−5^	3.86×10^−5^	2.15×10^−5^	5.57×10^−5^
Large Bud	3	1.09×10^−4^	7.30×10^−5^	1.79×10^−4^	1.88×10^−4^	1.33×10^−4^	3.24×10^−4^
Large Bud	4	6.08×10^−5^	4.11×10^−5^	1.07×10^−4^	3.40×10^−5^	2.42×10^−5^	6.09×10^−5^
Large Bud	5	8.85×10^−5^	6.14×10^−5^	1.53×10^−4^	9.57×10^−5^	5.84×10^−5^	1.43×10^−4^
Large Bud	6	2.47×10^−5^	1.32×10^−5^	4.10×10^−5^	2.07×10^−5^	1.13×10^−5^	3.43×10^−5^
Large Bud	7	5.10×10^−5^	3.18×10^−5^	8.28×10^−5^	3.13×10^−5^	2.15×10^−5^	5.64×10^−5^
Large Bud	8	3.48×10^−5^	2.28×10^−5^	5.83×10^−5^	2.51×10^−5^	1.44×10^−5^	4.02×10^−5^
Large Bud	9	2.14×10^−5^	1.32×10^−5^	3.73×10^−5^	2.63×10^−5^	1.59×10^−5^	4.44×10^−5^
Large Bud	10	1.73×10^−5^	1.18×10^−5^	3.55×10^−5^	1.79×10^−5^	1.13×10^−5^	3.30×10^−5^
Large Bud	11	9.80×10^−5^	6.60×10^−5^	1.66×10^−4^	4.11×10^−5^	2.70×10^−5^	6.95×10^−5^
Large Bud	12	1.10×10^−5^	6.32×10^−6^	2.03×10^−5^	6.37×10^−6^	3.75×10^−6^	1.44×10^−5^
Large Bud	13	4.95×10^−5^	3.53×10^−5^	8.82×10^−5^	2.45×10^−5^	1.60×10^−5^	4.24×10^−5^
Large Bud	14	1.97×10^−5^	1.19×10^−5^	3.40×10^−5^	1.37×10^−5^	8.18×10^−6^	2.39×10^−5^
Large Bud	15	2.05×10^−5^	1.13×10^−5^	3.49×10^−5^	1.64×10^−5^	9.06×10^−6^	2.75×10^−5^
Large Bud	16	8.27×10^−6^	4.05×10^−6^	1.59×10^−5^	1.34×10^−5^	6.78×10^−6^	2.32×10^−5^
Large Bud	17	3.70×10^−5^	2.20×10^−5^	6.06×10^−5^	1.67×10^−5^	9.42×10^−6^	2.72×10^−5^

Our V_M_/V_E_ estimates are lower than those of previous studies measuring a variety of phenotypes in a variety of organisms, which tended to report V_M_/V_E_ values between 10^−4^ and 5×10^−2^ [34]. Future experiments can address why this discrepancy exists. Our results might reflect a genuinely restricted range of mutational variance for this suite of traits in this organism (compared to other traits in multicellular organisms in particular). Alternatively, it must be considered that V_M_/V_E_ might increase with more precise measurements of V_E_, which is necessarily a combination of actual biological variation within lines and variation in measurement. Decreased measurement variance could be achieved, for example, by more precisely staging cells (reducing the variance in cell-cycle stage at which cells are measured).

## Discussion

Previous studies have demonstrated the release of cryptic genetic variation after a genetic or environmental perturbation [2], [9]–[16], [20]–[29]. This release is often conflated with a breakdown in mutational robustness [8]. However, the release of cryptic genetic variation is not a reliable indicator of mutational robustness when the genetic backgrounds that are studied have been subject to artificial or natural selection, as was the case in all prior studies [8]. Our study compares, for the first time, the relative mutational robustness conferred by two alleles, in a panel of genetic backgrounds that had accumulated naturally occurring mutations with minimal selection. We find that MA lines that are HTZ1+ are not more robust to new mutations than lines that are HTZ1−, as the two genotypes display similar extents of morphological variation across lines. Nevertheless, we find strong evidence of epistasis between HTZ1 and new mutations, manifest as a significant interaction between HTZ1 genotype and line. We also find strong evidence corroborating our previous finding [41] that HTZ1 deletion increases within-line variation. Taken together, our results indicate that wild-type HTZ1 function increases robustness to microenvironmental variation but not to mutations.

Theoretical studies have tended to predict congruence between robustness mechanisms, or in other words that mechanisms contributing to robustness to one source of variation will contribute to robustness to other sources [2], [42], [43]. The results presented here do not support this conclusion, at least with regard to HTZ1. Additional doubt has been cast on the congruence hypothesis by studies in Drosophila [15] and *E. coli* [51]. Future studies will be required to test whether the congruence hypothesis also does not hold for other genes or whether HTZ1 and these other cases are aberrations.

Our results highlight the importance of using MA lines for tests of mutational robustness. The lines used in this study have accumulated extensive genetic variation affecting cell morphology (Figure 2 and 3). It is reasonable to assume that had the HTZ1+ lines been exposed to stabilizing selection, then the phenotypic and genetic variation among HTZ1+ lines would have been reduced. However, replacing HTZ1+ with HTZ1− in this scenario would still likely reveal extensive phenotypic variation, because the mutations allowed to accumulate in the presence of HTZ1+ can have very different effects in its absence. We hypothesize that the findings of greater expression divergence between *S. cerevisiae* and *S. paradoxus* upon deletion of chromatin regulators (including HTZ1) [44] are due to the effect of selection, and that analysis of expression variation in wild-type and mutant MA lines would not reveal a suppressive effect of HTZ1 on expression change.

Our results are reminiscent of work on Hsp90 in yeast, even though that work was conducted with strains that had been subject to natural selection [23], [52]. Specifically, Hsp90 impairment in *S. cerevisiae* has been associated not only with increased between-strain variation for some traits but also with suppression of variation for other traits. For example, wild-type HSP90 function is necessary for some drug-resistance mutations to have their effects [52]. In a larger survey [23], 102 genetically divergent yeast strains were analyzed for their ability to grow in a variety of conditions, with wild-type and reduced levels of HSP90. A QTL analysis of HSP90-dependent traits showed that 44 HSP90-dependent growth QTLs were present at wild-type HSP90 levels and 63 HSP90-dependent growth QTLs were present at reduced HSP90 levels [23]. These findings have led to the description of Hsp90 as both a “capacitor” of phenotypic variation (suppressing the effects of genetic variants unless impaired) and a “potentiator” of phenotypic variation (permitting the effects of genetic variants unless impaired) [12], [23], [53]. Our work suggests that what is important about highly pleiotropic factors such as HSP90 and HTZ1 is not that they reduce robustness to mutations when impaired (which appears not to be true at least for HTZ1) but that they interact epistatically with mutations. That is, the effects of perturbing such a factor will be context- and phenotype-dependent, as will the factor's apparent role as capacitor or potentiator.

The present study adds to growing empirical support for the notion that pleiotropy and epistasis are widespread [54]. Understanding the evolutionary roles of HSP90, HTZ1 and other factors with large potential effects on phenotypic variation will require both more experimental analysis and better theoretical models of complex traits [54]. In particular, the evolutionary role of cryptic genetic variation remains poorly understood. Although cryptic genetic variation has historically been viewed as a product of mutational robustness, we lend empirical support to the argument [8] that mutational robustness is a side question. We suggest that there should be more focus on the cryptic variation itself and the mechanisms that reveal it, rather than on the putative cause of its existence.

## Materials and Methods

### Yeast strains and genetics

Diploid yeast MA lines were provided by David Hall [36], [37]. In brief, the lines originated from a haploid strain (a spore from a DBY4974/DBY4975 diploid) with genotype *ade2-101, lys2-801, his3-Δ200, leu2-3.112, ura3-52, Gal+, ho* [36]. This strain was made diploid using an *HO*-expressing plasmid, creating a diploid line homozygous at each locus except the mating-type locus, that served as the ancestor for all the MA lines [36]. Yeast with an *ade2* mutation build up a red metabolite during respiration, which acts as a visual marker that cells are not “petite” or respiration-deficient [55]. Cells lacking the red pigment were not passaged during the MA experiment to avoid accumulating mutations affecting mitochondrial function [36]. To produce the haploid MA lines for our study, we sporulated the diploid MA lines using a standard protocol [56]. For each MA line, a single non-petite spore of mating type a was chosen at random to be the representative HTZ1+ haploid of the line for the remainder of the experiment. The HTZ1 coding sequence was completely eliminated from representative haploid lines by homologous recombination of a linear fragment containing 471 base pairs of homology to the sequence immediately upstream of the HTZ1 coding sequence, 477 base pairs of homology to the sequence immediately downstream of the HTZ1 coding sequence, and, in between these, the URA3 selectable marker, using standard techniques [57]. PCR analysis of transformants capable of growth on medium lacking uracil confirmed the correct incorporation of the linear fragment and the complete absence of the HTZ1 coding sequence. A single confirmed transformant from each line was chosen as the representative HTZ1− line for morphological analysis. Ultimately, 79 line pairs were used for our experiments (listed in Table S5).

Although the MA lines have the *ade2-101* mutation, which causes colonies to be red, we noted the appearance of white or sectored colonies while propagating some of the lines. The loss of red pigmentation can indicate the presence of a petite mutation, a mutation that restores ADE2 function, or an epigenetic mechanism affecting the adenine pathway. The frequency of white-to-red switches and red-to-white switches, as well as sizes of the sectors or colonies, suggested an epigenetic factor was responsible. We hypothesize that the epigenetic factor is [PSI+], a prion form of the translation-termination factor SUP35, because [PSI+] causes increased read-through of stop codons [58] and *ade2-101* is a premature stop-codon mutation [59]. Further analysis of this hypothesis will be presented elsewhere. For the purposes of the experiments presented here, we performed analyses two ways: by ignoring the sectoring behavior and by restricting our attention to only the 43 lines that stably maintained red color. These analyses yielded substantially similar conclusions so for simplicity we report the analysis for the full set of lines throughout the paper, while noting in some important places the results for the restricted analysis as well. The identities of the 43 stable-red lines are indicated in Table S5.

### Microscopy and image analysis

Cell morphology was measured in many cells from each pair of MA lines (HTZ1+ and HTZ1−) using a microscopy-based phenotyping assay [47], adapted for a 96-well plate format rather than for individual glass slides. The original assay used three fluorescent signals to measure cell morphology. However, only two, ConcanavalinA-FITC, which stains the cell surface, and DAPI, which stains the nucleus, were used in this study. A previous study indicated that the third signal, rhodamine-phalloidin, did not add significant information to the analysis of morphological variance [41], and was consequently excluded from this analysis. Cells were grown to mid-log phase in 96-well culture plates. Cells were then fixed with 4% para-formaldehyde, stained with 250 ug/mL ConcanavalinA-FITC, and plated into a 96-well glass-bottom microscope plate in mounting medium containing an anti-fade agent (Vectashield or a 5 µM p-phenylenediamine/glycerol solution) and 70 ng/mL DAPI. Plates were imaged using an inverted fluorescent compound microscope (Nikon TE-2000). Seventy-five random non-overlapping images were acquired per well with a Qimaging FireWire camera and NIS Elements software. Because CalMorph processes jpeg image files of particular pixel dimensions, one raw 16-bit 1392×1040 tiff image was split in to two 696×520, 8-bit jpeg images. A minor background correction using the imadjust function in matlab was done to avoid discrepancies in staining quality. This adjustment did not significantly alter raw data values (data not shown). Images were then analyzed using CalMorph software, developed specifically for this assay [47].

### Statistics and data analysis

Raw cell measurements from CalMorph were analyzed using the R programming environment. CalMorph splits cells into three types: cells with no bud, a small bud, and a large bud. Each type has a unique set of morphological phenotypes and is therefore analyzed separately throughout this study. To allow for comparisons between CalMorph phenotypes of cells of the same type, raw values were transformed using the Box-Cox method [60] from the car package in R, and standardized to have a mean of zero and a standard deviation of one. The Box-Cox transformation method uses maximum likelihood to determine which among a family of power transformations produces a phenotypic distribution that approximates normality best [60]. Previous studies using CalMorph data have used this transformation [47], [61]–[63]. If a phenotype did not show an approximately normal distribution on a qq-plot after this procedure, it was excluded (the phenotypes used in the analysis are shown in Table S6). Only cells without missing values for each remaining phenotype were included in the final analysis.

The phenotypes measured by CalMorph are not completely independent [41], so principal component analysis (PCA) was used to eliminate redundant measures. PCA was performed on real and randomly permuted data (where raw values for a given trait were reassigned to a randomly chosen cell). Principal components capturing greater variance than the random expectation were used in downstream analyses. Performing PCA separately on HTZ1+ or HTZ1− cells yielded principal components with highly correlated loadings.

To confirm that our choice of PCA for eliminating redundancy did not unexpectedly bias results, we performed alternative analyses using partitioning around medoids (PAM), a variant of k-means clustering, as well. PAM clusters phenotypes by similarity and designates a “medoid,” the phenotype that is most representative of the other phenotypes in the cluster. PAM was used previously to eliminate redundancies in CalMorph-generated data [41]. Normalized and standardized data were clustered using the pam function of the cluster package in R. For each cell type, the number of significant principal components was used to determine the number of clusters used in the final analysis.

Variance-component estimates from linear models were obtained using Markov chain Monte Carlo sampling, with the R package MCMCglmm [64]. Highest posterior density interval estimates were obtained from the MCMC samples (or functions of these samples) using the HPDinterval function in the coda package [64]. The linear model specified two within-strain variances, one each for HTZ1+ and HTZ1−, using the “idh” variance structure for the residual variance. Likewise, it specified two between-strain variances, and also a genetic covariance, using the “us” variance structure. Inverse-Wishart priors were used. Parameter-expanded priors, which are suggested to have better properties when variance components are close to 0, were also tried, with negligible difference in results. Markov chains were run with a burn-in period of 6000, and samples were stored at intervals of 15 iterations for 30000 total iterations. Chain lengths were kept relatively short because of the large number of models that were run, so it was important to verify that the chains were well mixed. We did so by examining the autocorrelation in parameter estimates between successive stored samples, which was very close to 0 after the burn-in period. We also ran longer chains for a small subset of models, and found negligible effects on the parameter estimates.

To test for an interaction between line and genotype, linear mixed models were fitted using the lmer function from the lme4 package in R. Genotype (HTZ1+ or HTZ1−) was modeled as a fixed effect, whereas MA line and a line-by-genotype interaction term were modeled as random effects. Note that lmer assumes a single within-line variance and a single between-line variance.

The genotype-by-line interaction variance was partitioned into components that correspond to line crossing and line spreading by this formula:
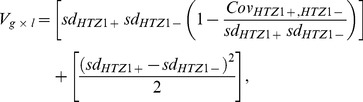
(1)where sd_HTZ1+_ and sd_HTZ1−_ are the HTZ1+ and HTZ1− between-line standard deviations, respectively. This formula is identical to that given for V_g×l_ previously [30], except r_HTZ1+,HTZ1−_, a term representing the correlation of line means, is replaced by 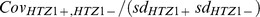 [65]. The crossing of line means is represented by the first bracketed term in Equation 1, and the spreading of line means is represented by the second bracketed term in Equation 1. See Text S1 for estimation of the crossing and spreading terms when models were fit by restricted maximum likelihood.

Estimates of mutational variance were made using the equation [66]:
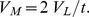
V_L_ is the between-line variance, for one or the other HTZ1 genotype, estimated by MCMC, discussed above. t, the number of generations, was estimated as 2062 [37]. Note that this V_M_ is the mutational variance of the haploid lines in which we actually measured phenotypes, not of the diploid lines from which they derive.

At least 150 cells were measured of each cell type from each line. This number was chosen based on a sub-sampling analysis, where an increasing number of cells was randomly drawn (without replacement) and used to calculate a mean and standard deviation value. Each of these estimates tended to converge when sampling more than 150 cells, suggesting this number is sufficient to adequately estimate the line means and within-line variances for a given morphological trait (see Figure S7 for an example trait).

## Supporting Information

Figure S1 Scree plots from principal component analysis. The plots show the percentage of variance explained by each principal component, for all three cell types: (A) unbudded cells, (B) cells with small buds, and (C) cells with large buds. The black circles represent principal components obtained by PCA on real data. Red circles represent principal components from data randomly permuted within phenotypes before PCA was performed. Only principal components that explain more variance than the random expectation are studied further in this paper.
(PDF)

Figure S2 Correcting effect of median on MAD by lowess regression. (A) Line MADs plotted against medians for principal component 1 of the unbudded cell type. The black line is the lowess curve. Red circles are HTZ1− lines and blue circles are HTZ1+ lines. (B) The same data from A, with the residuals to the lowess curve, instead of the MADs, plotted against the median.
(PDF)

Figure S3 Mean principal component values of HTZ1+ and HTZ1− lines for each principal component in each cell class: (A) unbudded cells, (B) cells with small buds, and (C, D) cells with large buds. Each line connects an HTZ1+ MA line with its HTZ1− derivative. The means and standard deviations of line means are indicated by the black circles and bars. The mean of the ancestral strain is shown to the left in each plot.
(PDF)

Figure S4 Mean principal component values of HTZ1+ and HTZ1− lines, as in Figure 2. Values are scaled so that the distributions of line means have equivalent mean and standard deviation, to show the extent of line crossing.
(PDF)

Figure S5 Average silhouette widths for PAM with different numbers of clusters for each cell class: (A) unbudded cells, (B) cells with small buds, and (C) cells with large buds. MAX indicates the number of clusters that maximizes the mean silhouette width. PC indicates the number of significant principal components calculated by PCA (see Materials and Methods).
(PDF)

Figure S6 Estimates of mean and standard deviation of line means for principal component 1 for unbudded cells. A random subsample containing the given number of MA line pairs (horizontal axis) was used. Red represents HTZ1− values and black represents HTZ1+ values.
(PDF)

Figure S7 Trait mean and median absolute deviation (MAD) as a function of sample size. Estimates of unbudded cell area mean (top) and MAD (bottom) with increasing sample size. Samples of the given size were drawn randomly from MA line 12, with HTZ1 intact.
(PDF)

Table S1 Analysis of between-line variance. P-values are given for Levene's test of differences in between-line variance (column 3), likelihood-ratio test for significance of genotype-by-line interaction term (column 4) and likelihood-ratio test for significance of genotype-by-line interaction term after scaling data to remove spreading (column 7) for each principal component (PC). Estimated percentages of total interaction variance explained by line crossing (column 5) and line spreading (column 6) are also given. * = PC for which HTZ1− lines have a significantly greater between-line variance than HTZ1+ lines (p<0.05 by Levene's test). † = PC for which HTZ1+ lines have a significantly greater between-line variance than HTZ1− lines (p<0.05 by Levene's test).
(PDF)

Table S2 Estimates of within-line variance, along with 95% credible intervals (CrI), for HTZ1+ lines and HTZ1− lines, derived from MCMC. * = medoid for which the CrI of the difference between HTZ1+ and HTZ1− in the within-line variance estimates does not overlap 0, and for which the HTZ1+ within-line variance is lower than that of HTZ1−.
(PDF)

Table S3 Estimates of between-line variance, along with 95% credible intervals (CrI), for HTZ1+ lines and HTZ1− lines, derived from MCMC. * = medoid for which the CrI of the difference between HTZ1+ and HTZ1− in the between-line variance estimates does not overlap 0, and for which the HTZ1+ between-line variance estimate is higher than that of HTZ1−. † = medoid for which the CrI of the difference between HTZ1+ and HTZ1− in the between-line variance estimates does not overlap 0, and for which the HTZ1+ between-line variance estimate is lower than that of HTZ1−.
(PDF)

Table S4 Analysis of between-line variance, using PAM instead of PCA for dimensional reduction. P-values are given for Levene's test of differences in between-line variance (column 3), likelihood-ratio test for significance of genotype-by-line interaction term (column 4) and likelihood-ratio test for significance of genotype-by-line interaction term after scaling data to remove spreading (column 7). Estimated percentages of total interaction variance explained by line crossing (column 5) and line spreading (column 6) are also given. * = medoid traits for which HTZ1− lines have a significantly greater between-line variance than HTZ1+ lines (p<0.05 by Levene's test). † = medoid traits for which HTZ1+ lines have a significantly greater between-line variance than HTZ1− lines (p<0.05 by Levene's test).
(PDF)

Table S5 List of MA lines used in this study. Lines that are classified as having a stable-red inheritance pattern are indicated with an X in the stability column (see text).
(PDF)

Table S6 Traits for each cell type measured by CalMorph and used in this study. Each trait is listed by the name used by CalMorph and is accompanied by a brief biological description of what that trait measures.
(PDF)

Text S1 Details of model-independent measures of within-line and between-line variance and linear mixed modeling.
(PDF)
