# Supplementary figures and images for: Histone Variant HTZ1 Shows Extensive Epistasis with, but Does Not Increase Robustness to, New Mutations
Source: PLoS Genet. 2013 Aug 22;9(8):e1003733. doi: 10.1371/journal.pgen.1003733 (PMC3749942; doi:10.1371/journal.pgen.1003733)

**A**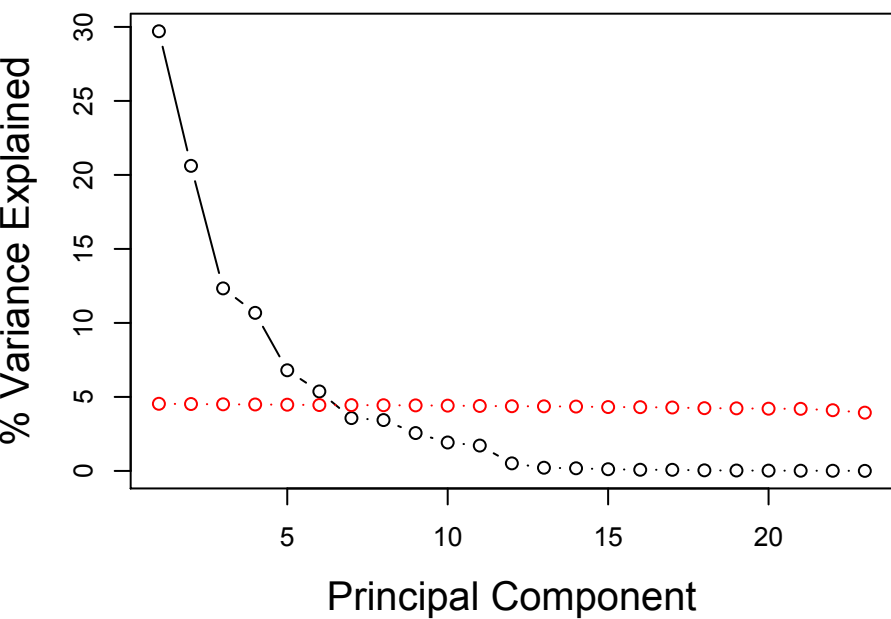**B**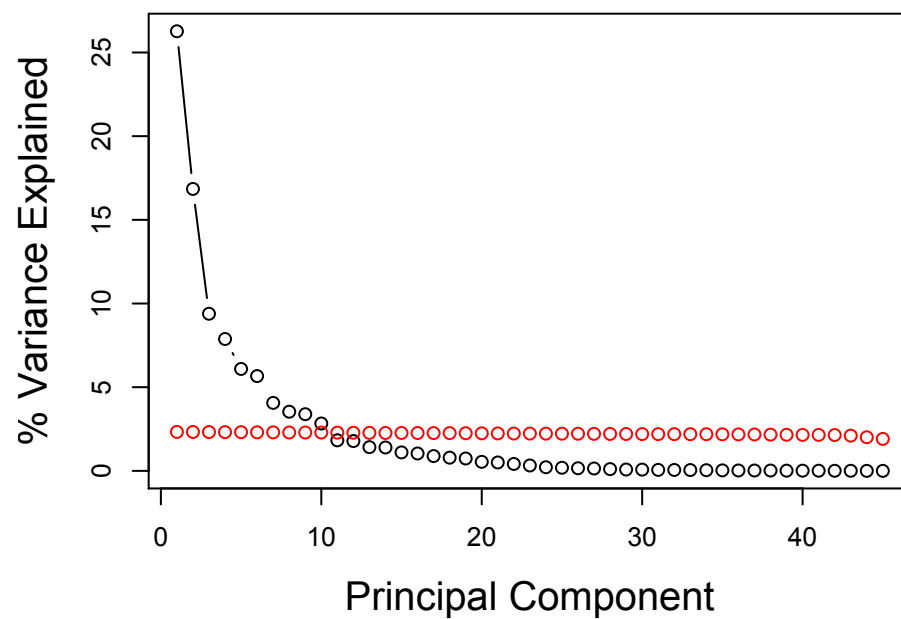**C**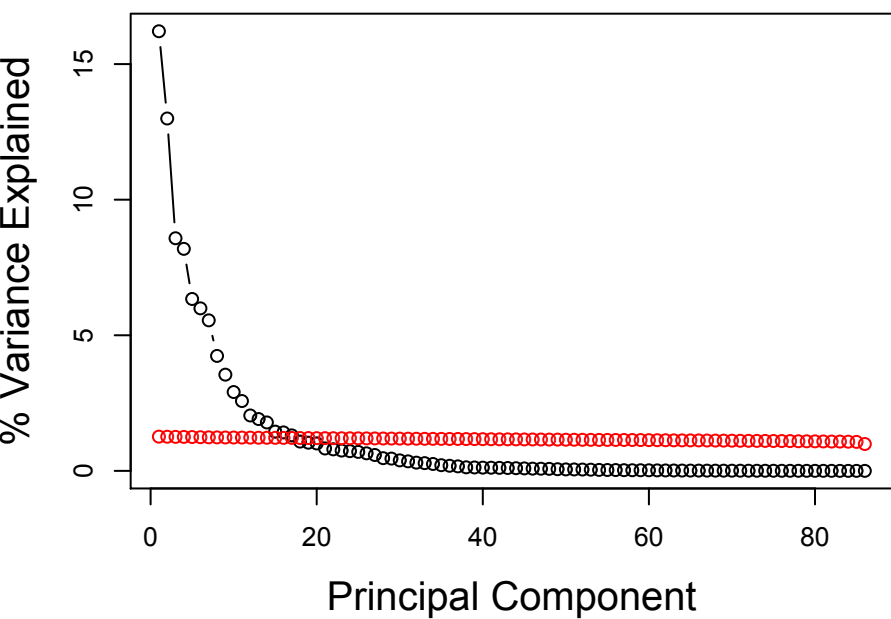

Supplement: Figure S1 — Scree plots from principal component analysis. The plots show the percentage of variance explained by each principal component, for all three cell types: (A) unbudded cells, (B) cells with small buds, and (C) cells with large buds. The black circles represent principal components obtained by PCA on real data. Red circles represent principal components from data randomly permuted within phenotypes before PCA was performed. Only principal components that explain more variance than the random expectation are studied further in this paper. (PDF) [file pgen.1003733.s001.pdf]

**A**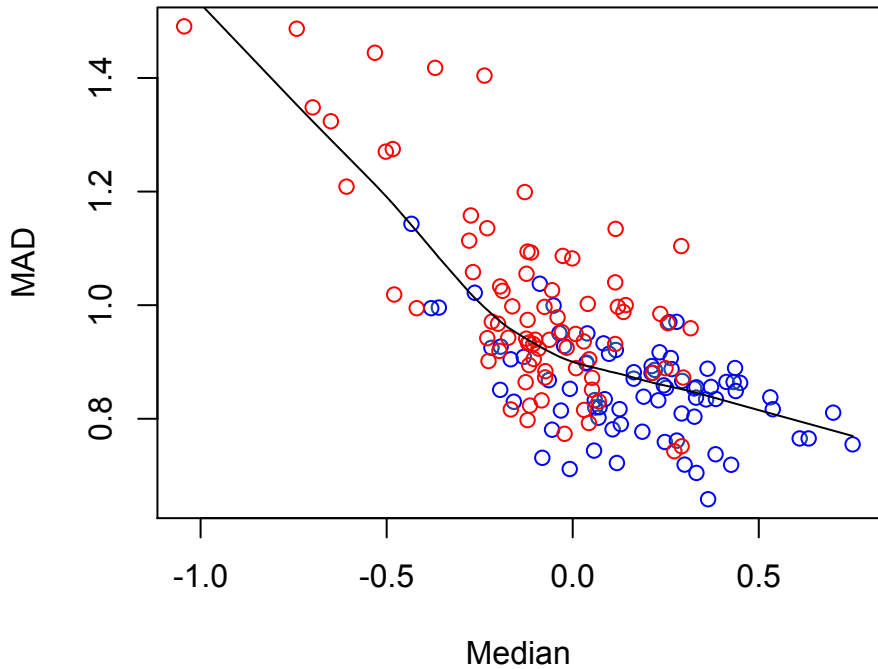**B**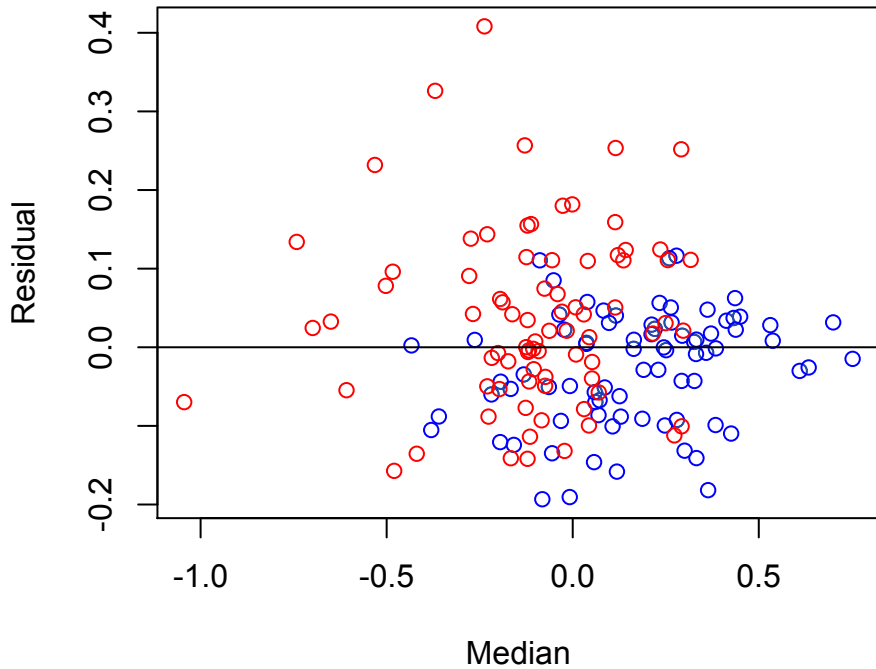

Supplement: Figure S2 — Correcting effect of median on MAD by lowess regression. (A) Line MADs plotted against medians for principal component 1 of the unbudded cell type. The black line is the lowess curve. Red circles are HTZ1− lines and blue circles are HTZ1+ lines. (B) The same data from A, with the residuals to the lowess curve, instead of the MADs, plotted against the median. (PDF) [file pgen.1003733.s002.pdf]

## A. No Bud

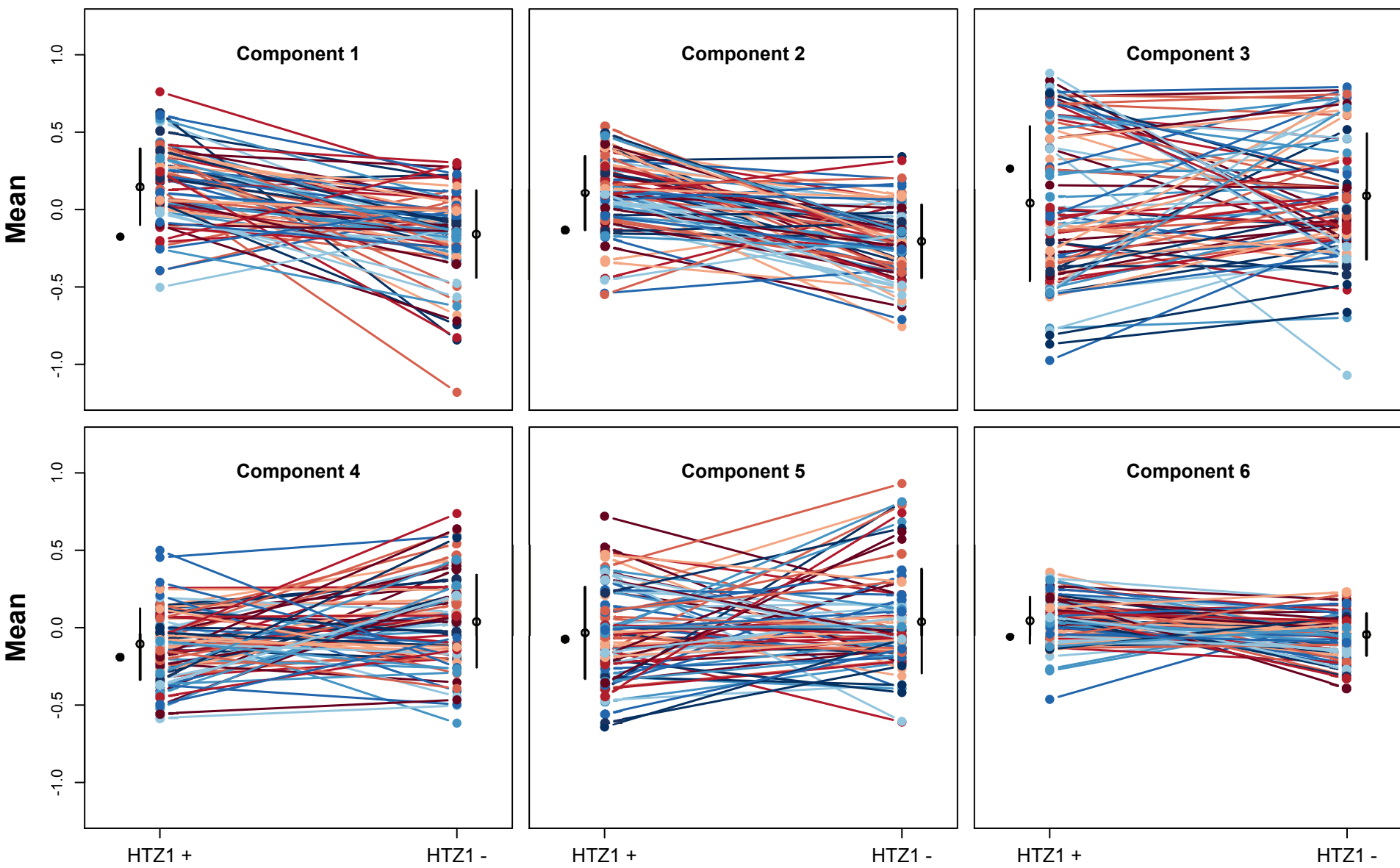

## B. Small Bud

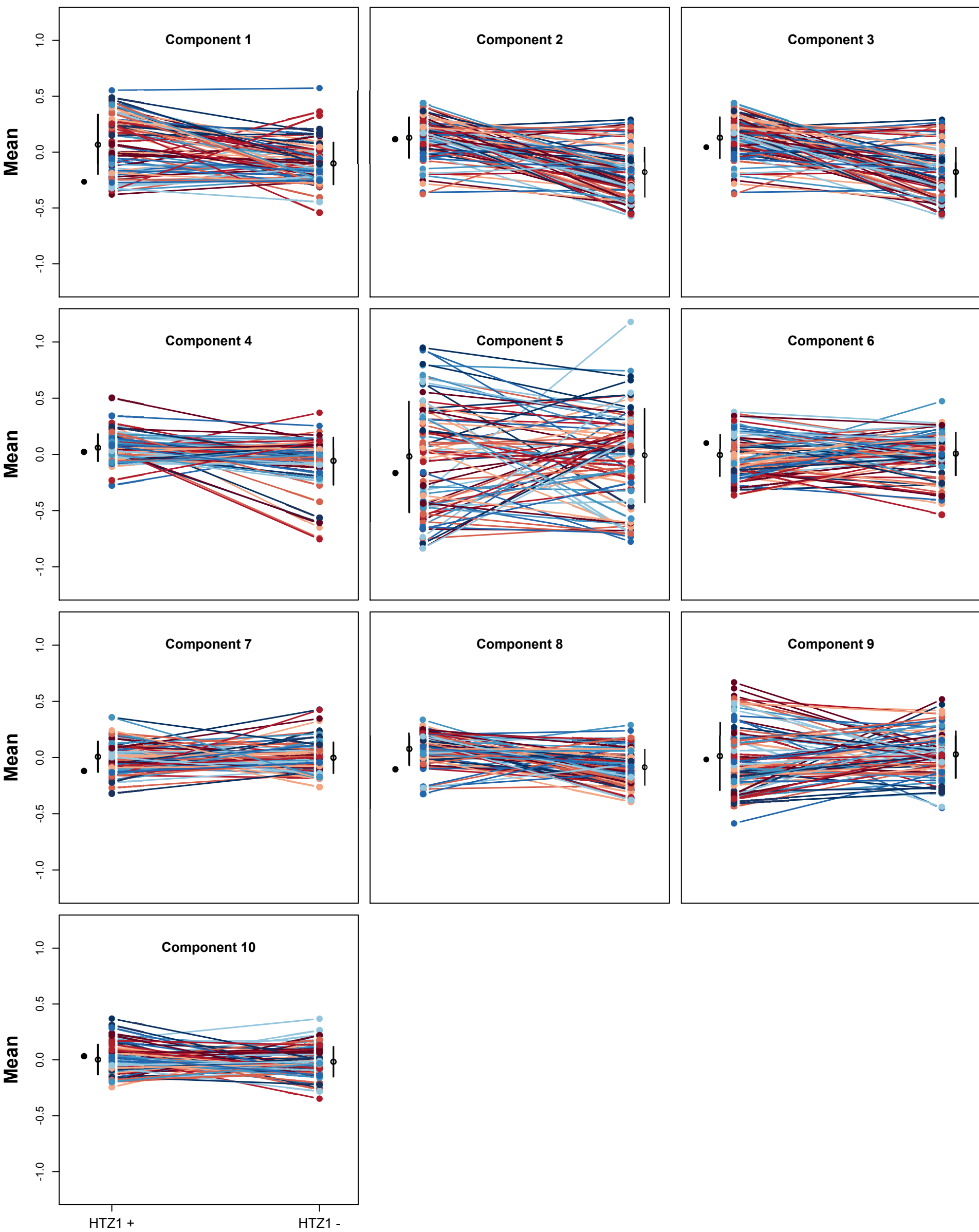

## C. Large Bud

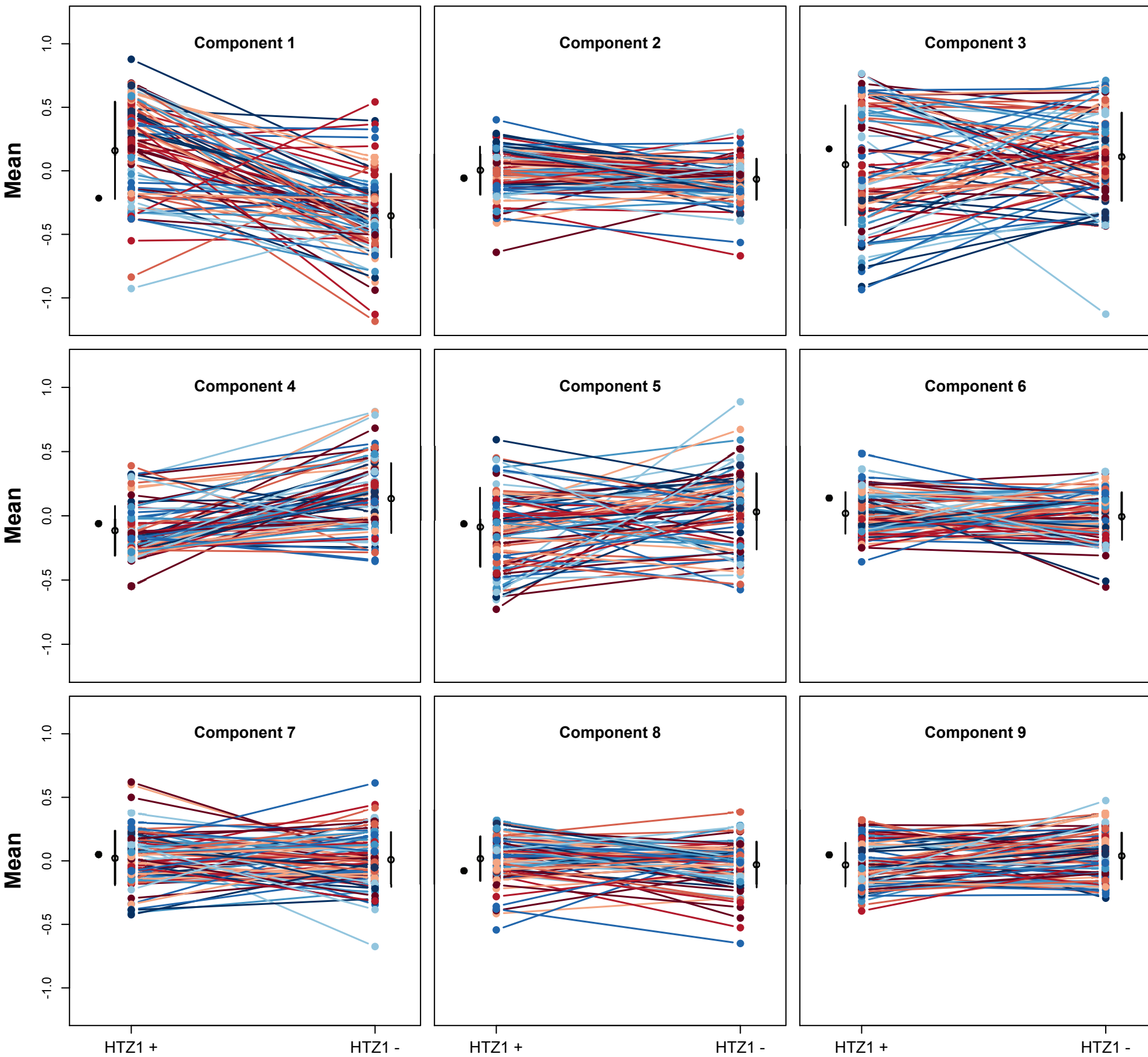

## D. Large Bud, Continued

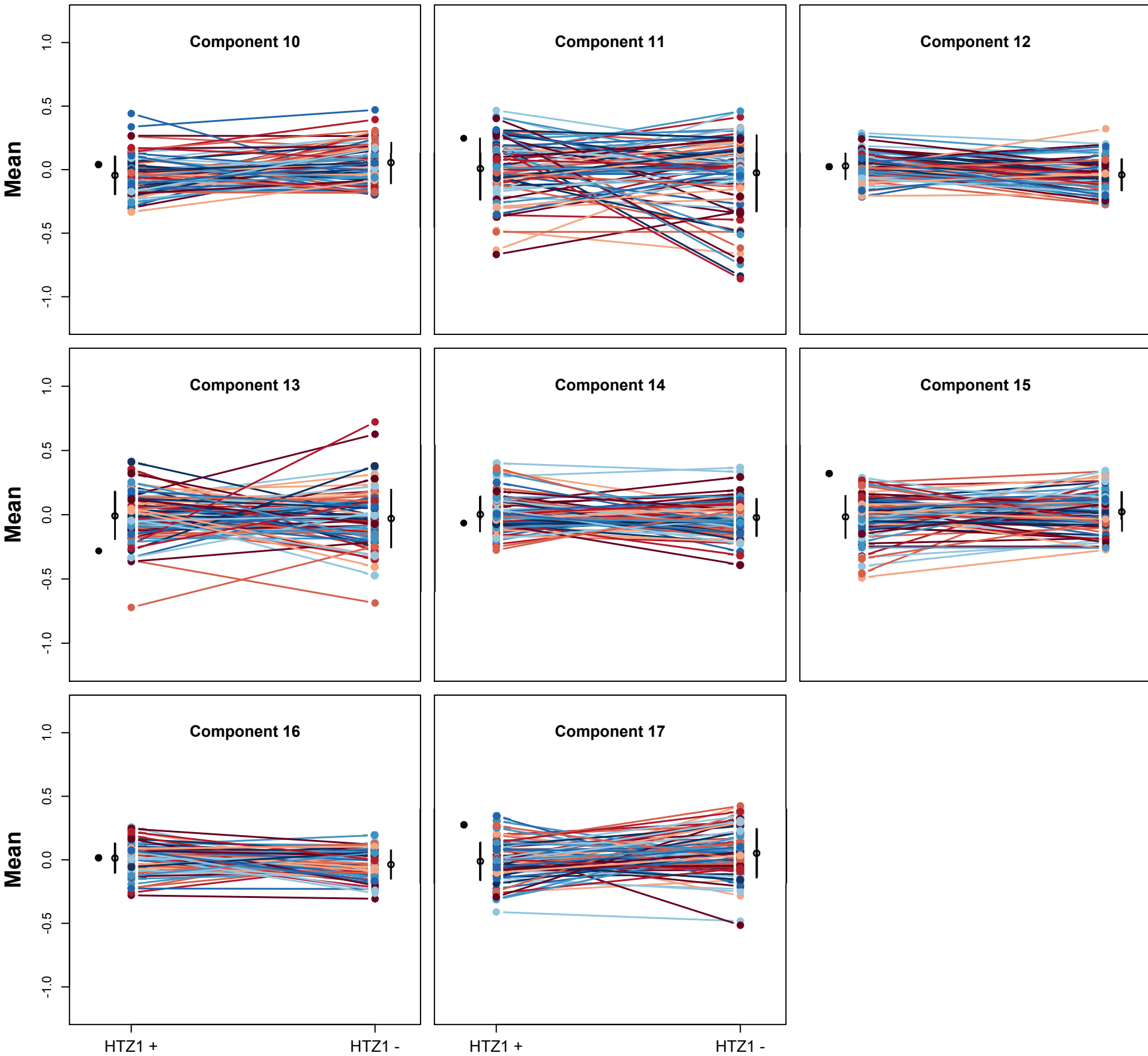

Supplement: Figure S3 — Mean principal component values of HTZ1+ and HTZ1− lines for each principal component in each cell class: (A) unbudded cells, (B) cells with small buds, and (C, D) cells with large buds. Each line connects an HTZ1+ MA line with its HTZ1− derivative. The means and standard deviations of line means are indicated by the black circles and bars. The mean of the ancestral strain is shown to the left in each plot. (PDF) [file pgen.1003733.s003.pdf]

## No Bud

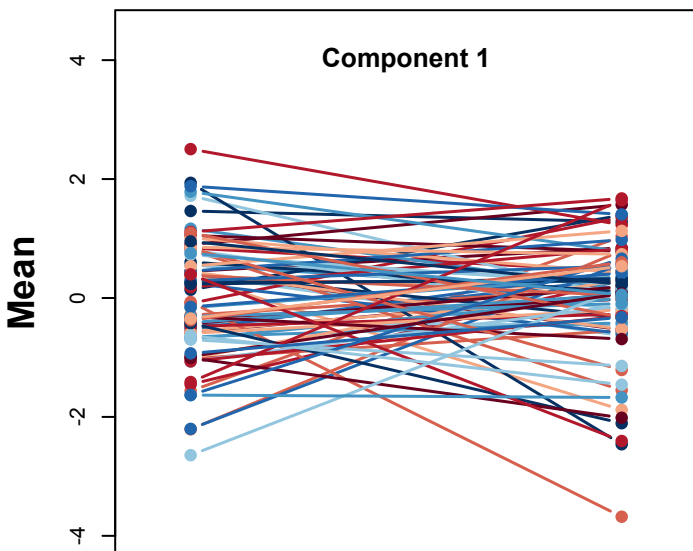

## Small Bud

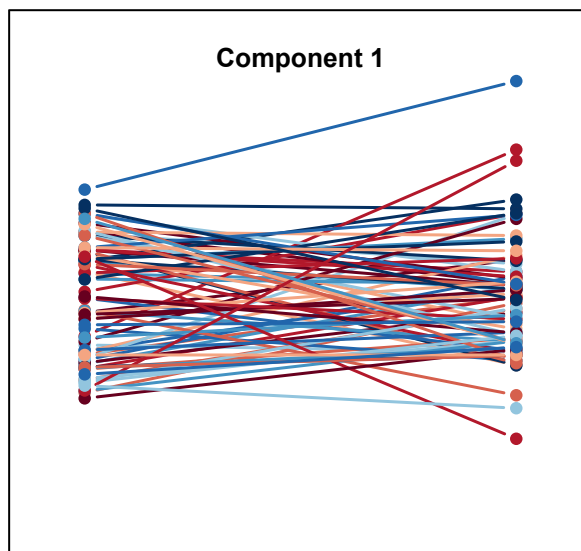

## Large Bud

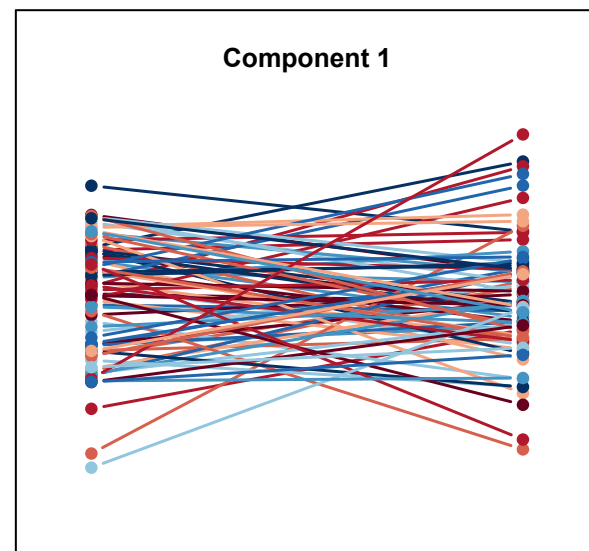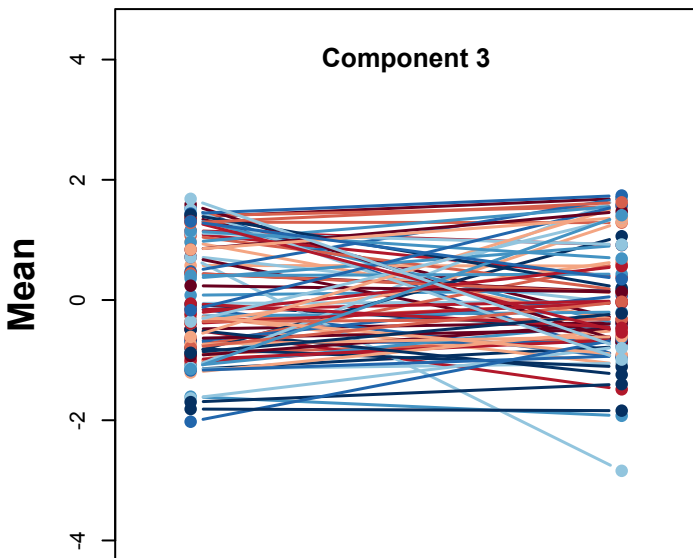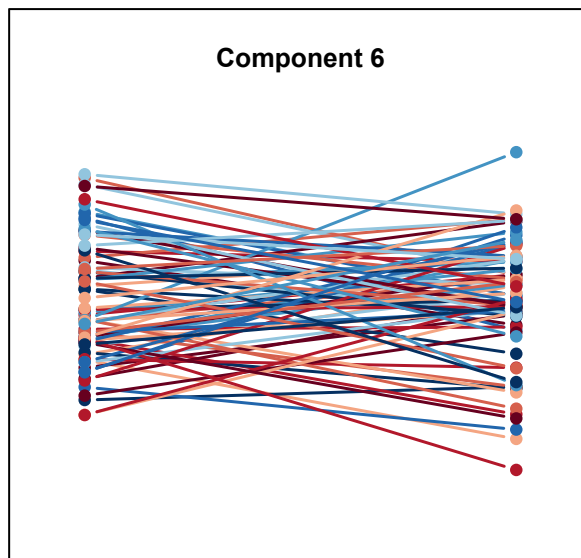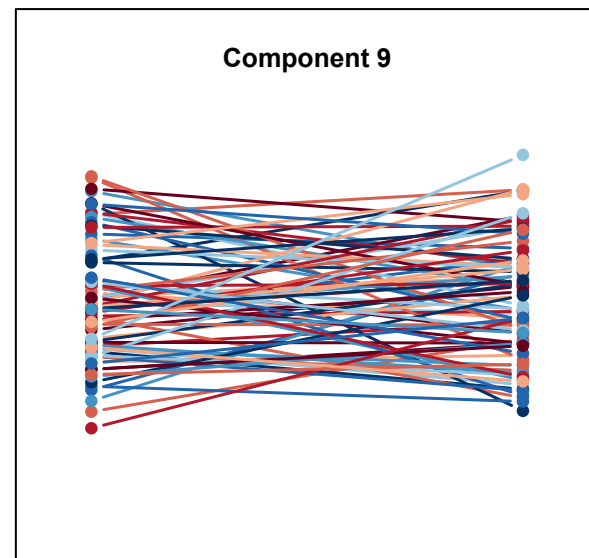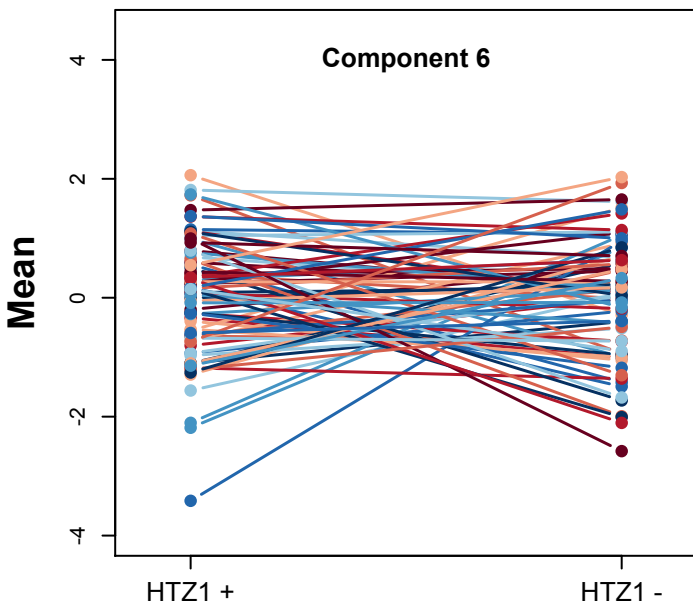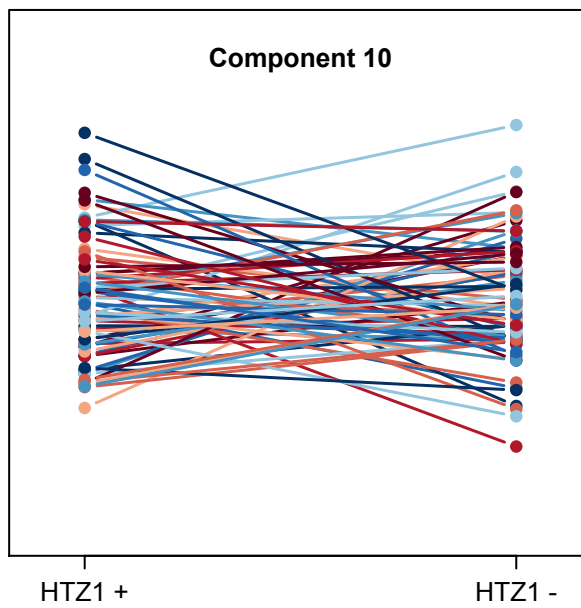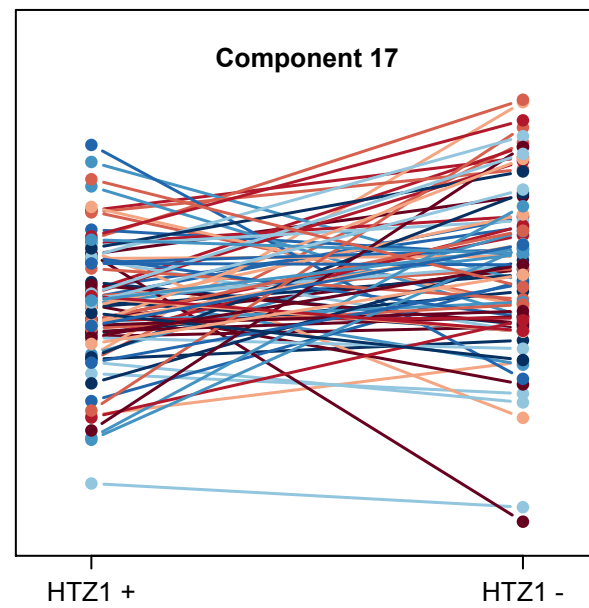

Supplement: Figure S4 — Mean principal component values of HTZ1+ and HTZ1− lines, as in Figure 2. Values are scaled so that the distributions of line means have equivalent mean and standard deviation, to show the extent of line crossing. (PDF) [file pgen.1003733.s004.pdf]

**A**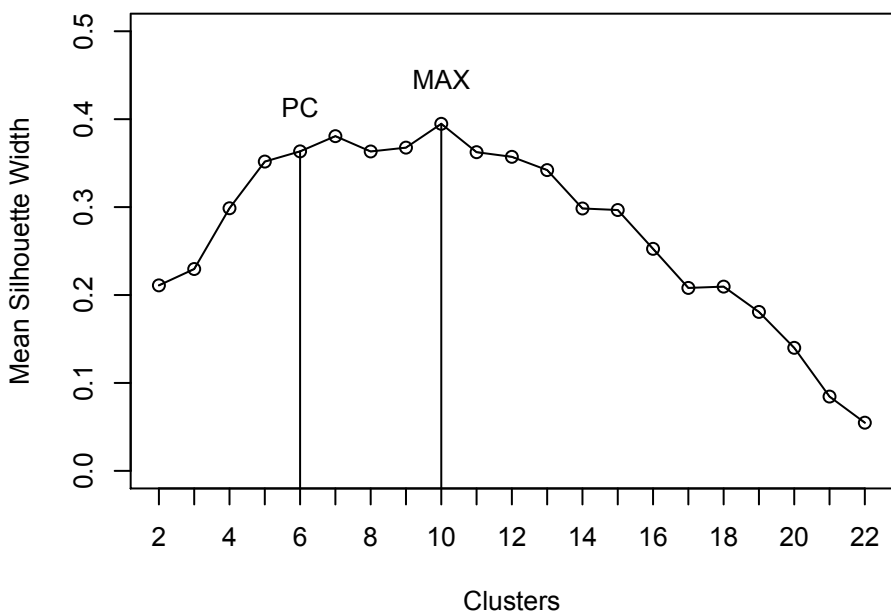**B**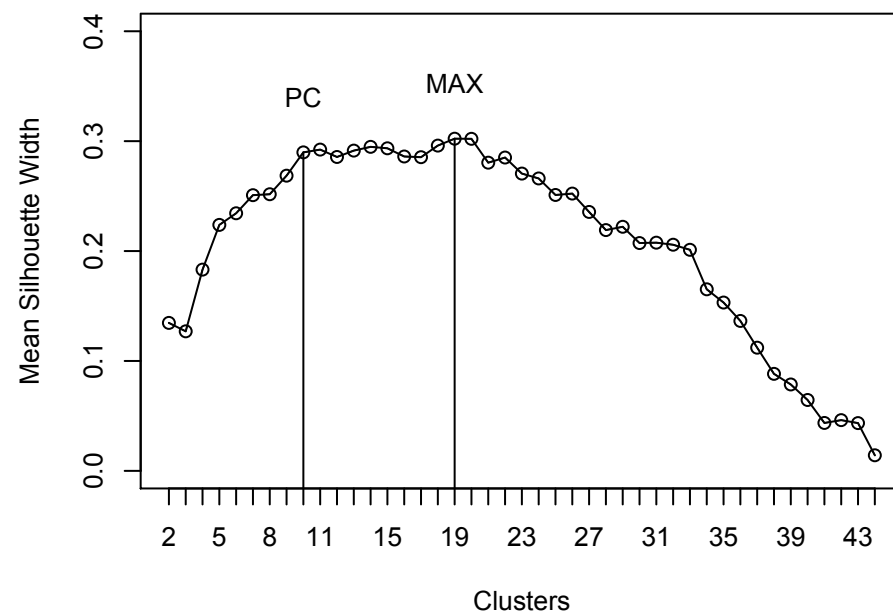**C**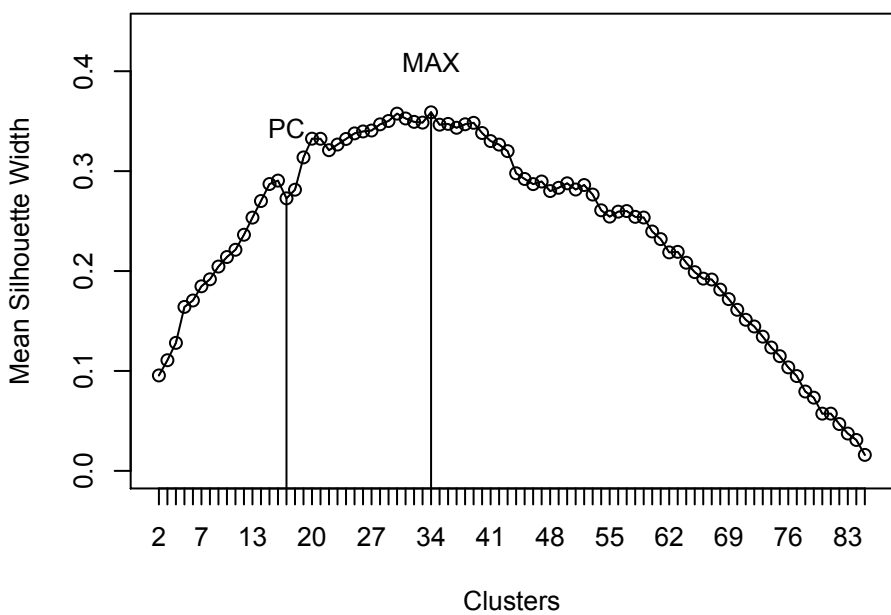

Supplement: Figure S5 — Average silhouette widths for PAM with different numbers of clusters for each cell class: (A) unbudded cells, (B) cells with small buds, and (C) cells with large buds. MAX indicates the number of clusters that maximizes the mean silhouette width. PC indicates the number of significant principal components calculated by PCA (see Materials and Methods). (PDF) [file pgen.1003733.s005.pdf]

Mean of line means

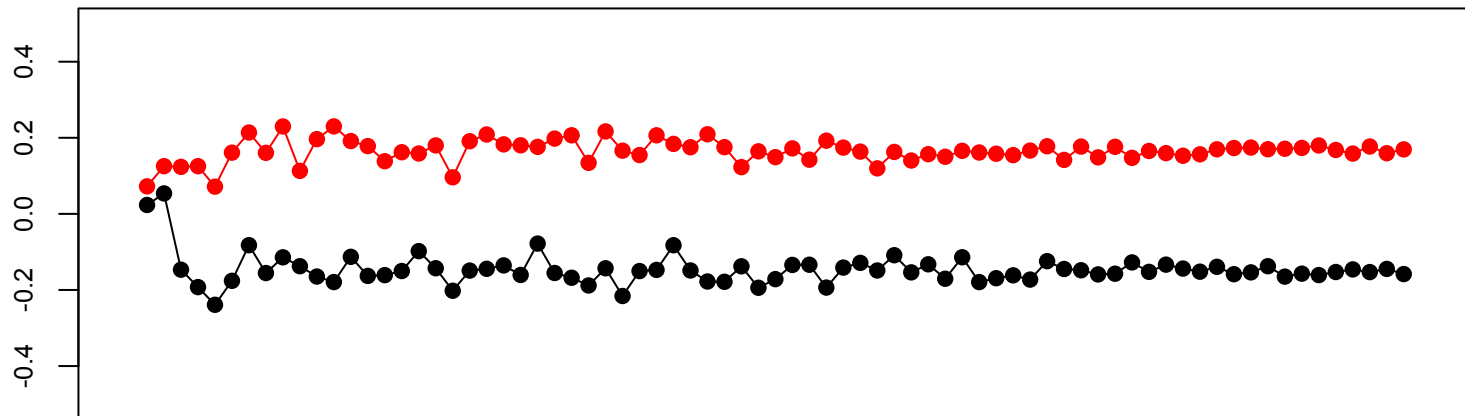

SD of line means

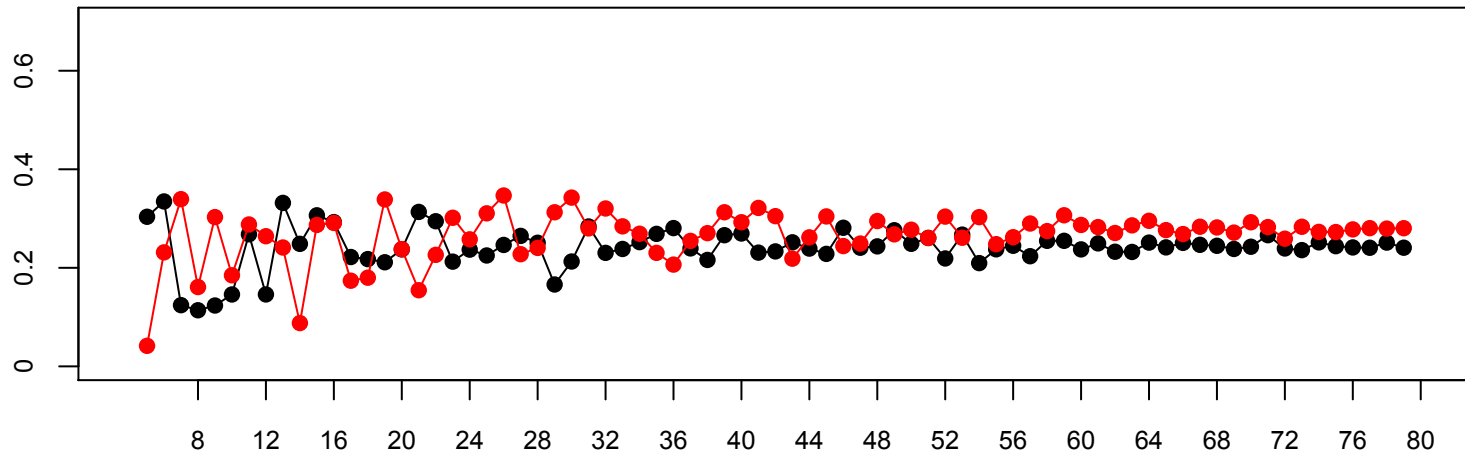

Number of Lines

Supplement: Figure S6 — Estimates of mean and standard deviation of line means for principal component 1 for unbudded cells. A random subsample containing the given number of MA line pairs (horizontal axis) was used. Red represents HTZ1− values and black represents HTZ1+ values. (PDF) [file pgen.1003733.s006.pdf]

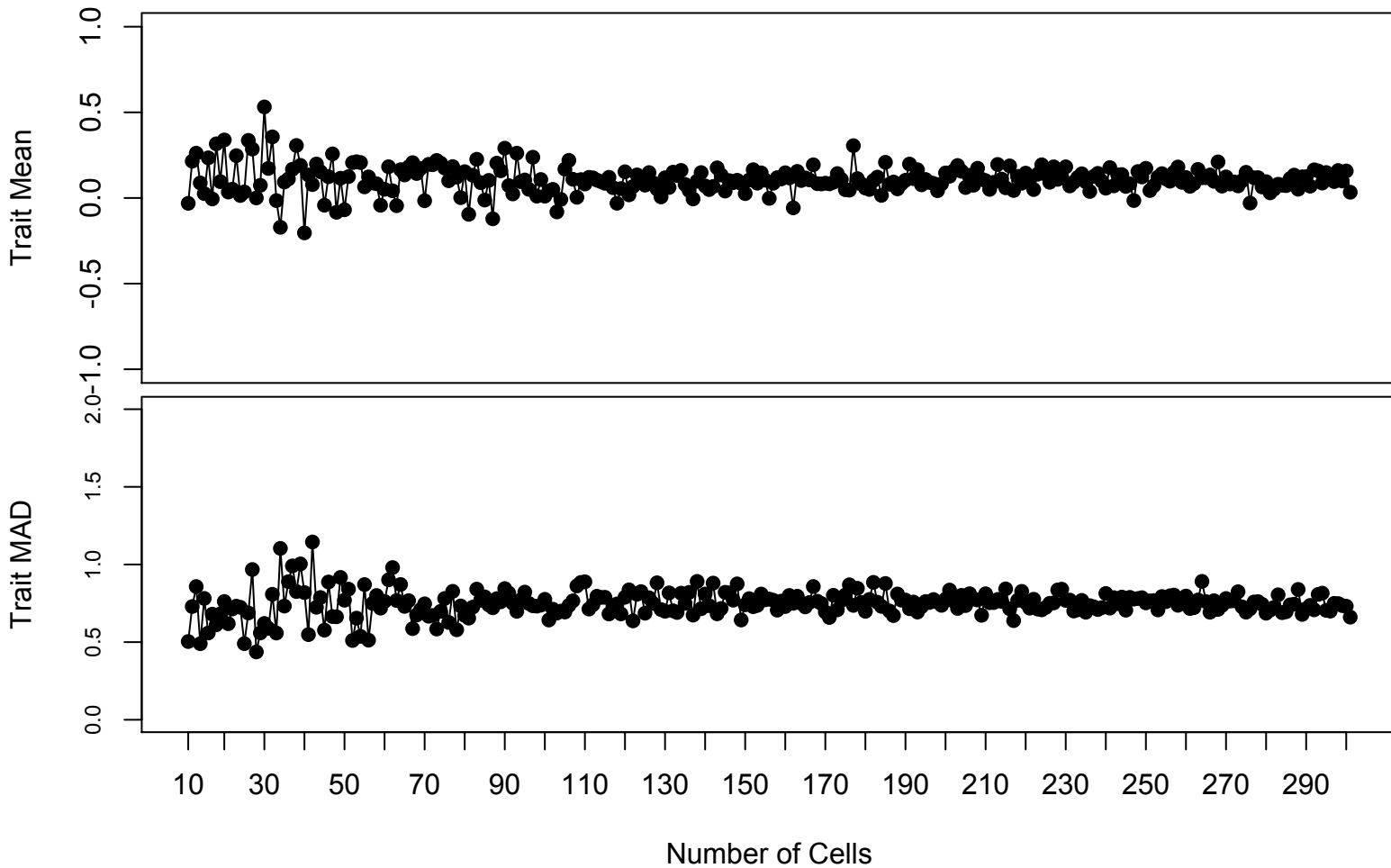

Supplement: Figure S7 — Trait mean and median absolute deviation (MAD) as a function of sample size. Estimates of unbudded cell area mean (top) and MAD (bottom) with increasing sample size. Samples of the given size were drawn randomly from MA line 12, with HTZ1 intact. (PDF) [file pgen.1003733.s007.pdf]
