# Supplementary material for: Histone Variant HTZ1 Shows Extensive Epistasis with, but Does Not Increase Robustness to, New Mutations
Source: PLoS Genet. 2013 Aug 22;9(8):e1003733. doi: 10.1371/journal.pgen.1003733 (PMC3749942; doi:10.1371/journal.pgen.1003733)
Supplement: Table S1 — Analysis of between-line variance. P-values are given for Levene's test of differences in between-line variance (column 3), likelihood-ratio test for significance of genotype-by-line interaction term (column 4) and likelihood-ratio test for significance of genotype-by-line interaction term after scaling data to remove spreading (column 7) for each principal component (PC). Estimated percentages of total interaction variance explained by line crossing (column 5) and line spreading (column 6) are also given. * = PC for which HTZ1− lines have a significantly greater between-line variance than HTZ1+ lines (p<0.05 by Levene's test). † = PC for which HTZ1+ lines have a significantly greater between-line variance than HTZ1− lines (p<0.05 by Levene's test). (PDF) [file pgen.1003733.s008.pdf]

Table S1. Analysis of between-line variance.

Cell Type	PC	P, Levene's Test	P, Interaction	Crossing	Spreading	Scaled P, Interaction
No Bud	1	0.8787	$<10^{-16}$	99.159	0.841	$<10^{-16}$
No Bud	2	0.8632	$<10^{-16}$	99.959	0.041	$<10^{-16}$
No Bud	3	0.0154 †	$<10^{-16}$	96.900	3.100	$<10^{-16}$
No Bud	4	0.0212 *	$<10^{-16}$	96.144	3.856	$<10^{-16}$
No Bud	5	0.8446	$<10^{-16}$	98.921	1.079	$<10^{-16}$
No Bud	6	0.3772	$<10^{-16}$	99.472	0.528	$<10^{-16}$
Small Bud	1	1.22×10^{-6} †	$<10^{-16}$	91.490	8.510	$<10^{-16}$
Small Bud	2	0.0177 *	$<10^{-16}$	97.823	2.177	$<10^{-16}$
Small Bud	3	0.0550	$<10^{-16}$	96.140	3.860	$<10^{-16}$
Small Bud	4	0.0222 *	$<10^{-16}$	83.021	16.979	$<10^{-16}$
Small Bud	5	0.0306 †	$<10^{-16}$	98.020	1.980	$<10^{-16}$
Small Bud	6	0.5907	$<10^{-16}$	99.911	0.089	$<10^{-16}$
Small Bud	7	0.5735	$<10^{-16}$	99.981	0.019	$<10^{-16}$
Small Bud	8	0.1639	$<10^{-16}$	99.595	0.405	$<10^{-16}$
Small Bud	9	0.0020 †	$<10^{-16}$	92.146	7.854	$<10^{-16}$
Small Bud	10	0.9733	$<10^{-16}$	99.968	0.032	$<10^{-16}$
Large Bud	1	0.0604	$<10^{-16}$	98.423	1.577	$<10^{-16}$
Large Bud	2	0.0310 †	$<10^{-16}$	96.739	3.261	$<10^{-16}$
Large Bud	3	0.0002 †	$<10^{-16}$	94.007	5.993	$<10^{-16}$
Large Bud	4	0.0013 *	$<10^{-16}$	92.424	7.576	$<10^{-16}$
Large Bud	5	0.5473	$<10^{-16}$	99.931	0.069	$<10^{-16}$
Large Bud	6	0.2051	$<10^{-16}$	99.002	0.998	$<10^{-16}$
Large Bud	7	0.9395	$<10^{-16}$	99.999	0.001	$<10^{-16}$
Large Bud	8	0.9261	$<10^{-16}$	99.850	0.150	$<10^{-16}$
Large Bud	9	0.3819	$<10^{-16}$	99.822	0.178	$<10^{-16}$
Large Bud	10	0.1434	$<10^{-16}$	99.215	0.785	$<10^{-16}$
Large Bud	11	0.3150	$<10^{-16}$	97.178	2.822	$<10^{-16}$
Large Bud	12	0.2298	8.12×10^{-14}	96.955	3.045	1.36×10^{-12}
Large Bud	13	0.3039	$<10^{-16}$	98.279	1.721	$<10^{-16}$
Large Bud	14	0.3951	$<10^{-16}$	99.855	0.145	$<10^{-16}$
Large Bud	15	0.9326	$<10^{-16}$	99.486	0.514	$<10^{-16}$
Large Bud	16	0.4699	4.36×10^{-13}	99.889	0.111	4.07×10^{-13}
Large Bud	17	0.0420 *	$<10^{-16}$	95.514	4.486	$<10^{-16}$
