# Supplementary material for: Histone Variant HTZ1 Shows Extensive Epistasis with, but Does Not Increase Robustness to, New Mutations
Source: PLoS Genet. 2013 Aug 22;9(8):e1003733. doi: 10.1371/journal.pgen.1003733 (PMC3749942; doi:10.1371/journal.pgen.1003733)
Supplement: Table S2 — Estimates of within-line variance, along with 95% credible intervals (CrI), for HTZ1+ lines and HTZ1− lines, derived from MCMC. * = medoid for which the CrI of the difference between HTZ1+ and HTZ1− in the within-line variance estimates does not overlap 0, and for which the HTZ1+ within-line variance is lower than that of HTZ1−. (PDF) [file pgen.1003733.s009.pdf]

Table S2. Estimates of within-line variance, along with 95% credible intervals (CrI), for HTZ1+ lines and HTZ1– lines, derived from MCMC.

Trait		HTZ1+	CrI lower bound HTZ1+	CrI upper bound HTZ1+	HTZ1–	CrI lower bound HTZ1–	CrI upper bound HTZ1–
C12.1_A	*	0.644	0.637	0.650	1.093	1.085	1.109
C115_A	*	0.822	0.814	0.831	0.930	0.921	0.942
D176_A	*	0.670	0.664	0.677	1.114	1.104	1.129
D16.1_A	*	0.660	0.653	0.666	0.750	0.742	0.759
D117_A	*	0.813	0.805	0.821	1.140	1.128	1.154
D148_A	*	0.912	0.904	0.923	1.013	1.001	1.025
C12.1_A1B	*	0.771	0.759	0.780	1.087	1.071	1.103
C107_A1B	*	0.880	0.870	0.894	1.102	1.084	1.115
C13_A1B	*	0.879	0.868	0.890	1.103	1.087	1.118
C109_A1B	*	0.967	0.956	0.981	1.006	0.990	1.019
D178_A1B	*	0.924	0.914	0.938	1.013	0.998	1.027
D16.3_A1B	*	0.828	0.819	0.841	1.135	1.120	1.153
D104_A1B	*	0.866	0.858	0.881	1.098	1.085	1.117
D110_A1B	*	0.848	0.838	0.860	1.097	1.086	1.117
D136_A1B	*	0.804	0.793	0.814	1.018	1.006	1.035
D170_A1B		0.916	0.902	0.926	0.930	0.916	0.944
C101_C	*	0.662	0.643	0.675	0.982	0.960	1.014
D166_C		0.955	0.928	0.974	0.978	0.949	1.002
D158_C	*	0.811	0.788	0.826	0.844	0.819	0.864
D185_C	*	0.828	0.810	0.848	1.067	1.038	1.095
C116_C	*	0.814	0.796	0.835	1.024	0.999	1.056
D103_C	*	0.844	0.826	0.868	1.156	1.131	1.195
C117_C	*	0.846	0.826	0.868	1.082	1.057	1.113
D176_C	*	0.913	0.891	0.934	1.042	1.010	1.065
D177_C	*	0.942	0.919	0.963	1.018	0.994	1.051
D193_C	*	0.946	0.916	0.961	1.030	0.997	1.054
D108_C	*	0.831	0.812	0.852	1.068	1.036	1.094
D109_C	*	0.964	0.945	0.992	1.017	0.988	1.044
D117_C	*	0.811	0.797	0.834	1.113	1.075	1.136
D121_C	*	0.927	0.906	0.952	1.049	1.019	1.076
D150_C	*	0.888	0.870	0.912	1.094	1.068	1.128
D163_C	*	0.944	0.924	0.970	1.049	1.020	1.078
D198_C	*	0.919	0.898	0.941	1.037	1.009	1.066
