# Supplementary material for: Histone Variant HTZ1 Shows Extensive Epistasis with, but Does Not Increase Robustness to, New Mutations
Source: PLoS Genet. 2013 Aug 22;9(8):e1003733. doi: 10.1371/journal.pgen.1003733 (PMC3749942; doi:10.1371/journal.pgen.1003733)
Supplement: Table S3 — Estimates of between-line variance, along with 95% credible intervals (CrI), for HTZ1+ lines and HTZ1− lines, derived from MCMC. * = medoid for which the CrI of the difference between HTZ1+ and HTZ1− in the between-line variance estimates does not overlap 0, and for which the HTZ1+ between-line variance estimate is higher than that of HTZ1−. † = medoid for which the CrI of the difference between HTZ1+ and HTZ1− in the between-line variance estimates does not overlap 0, and for which the HTZ1+ between-line variance estimate is lower than that of HTZ1−. (PDF) [file pgen.1003733.s010.pdf]

Table S3. Estimates of between-line variance, along with 95% credible intervals (CrI), for HTZ1+ lines and HTZ1– lines, derived from MCMC.

Trait		HTZ1+	CrI lower bound HTZ1+	CrI upper bound HTZ1+	HTZ1–	CrI lower bound HTZ1–	CrI upper bound HTZ1–
C12.1_A		0.0602	0.0495	0.0918	0.0842	0.0582	0.1133
C115_A	†	0.0649	0.0468	0.0885	0.1605	0.1081	0.2073
D176_A		0.0569	0.0439	0.0835	0.0632	0.0485	0.0932
D16.1_A	*	0.2798	0.2241	0.4132	0.1427	0.1121	0.2110
D117_A		0.0437	0.0310	0.0595	0.0377	0.0263	0.0510
D148_A	†	0.0149	0.0113	0.0221	0.0330	0.0236	0.0470
C12.1_A1B	*	0.0705	0.0498	0.0981	0.0410	0.0292	0.0573
C107_A1B		0.0170	0.0125	0.0255	0.0108	0.0078	0.0167
C13_A1B	†	0.0132	0.0089	0.0182	0.0252	0.0174	0.0339
C109_A1B	†	0.0072	0.0044	0.0100	0.0402	0.0304	0.0589
D178_A1B		0.0588	0.0411	0.0796	0.0717	0.0533	0.1025
D16.3_A1B	*	0.2864	0.2234	0.4164	0.1513	0.1062	0.2058
D104_A1B		0.0505	0.0377	0.0733	0.0392	0.0269	0.0523
D110_A1B		0.0264	0.0195	0.0376	0.0358	0.0240	0.0479
D136_A1B		0.0203	0.0135	0.0280	0.0250	0.0180	0.0361
D170_A1B		0.0044	0.0023	0.0066	0.0065	0.0042	0.0098
C101_C	*	0.0675	0.0513	0.0972	0.0413	0.0291	0.0568
D166_C		0.0171	0.0125	0.0257	0.0120	0.0078	0.0170
D158_C		0.0122	0.0087	0.0187	0.0229	0.0169	0.0340
D185_C		0.0093	0.0068	0.0151	0.0152	0.0105	0.0219
C116_C		0.0069	0.0047	0.0109	0.0416	0.0275	0.0535
D103_C		0.0070	0.0045	0.0103	0.0396	0.0307	0.0597
C117_C		0.0171	0.0118	0.0240	0.0111	0.0079	0.0169
D176_C		0.0252	0.0190	0.0376	0.0374	0.0274	0.0534
D177_C	†	0.0668	0.0491	0.0924	0.1144	0.0870	0.1654
D193_C	*	0.1062	0.0741	0.1398	0.0334	0.0243	0.0471
D108_C		0.0627	0.0433	0.0828	0.0714	0.0529	0.0994
D109_C	*	0.2905	0.2239	0.4213	0.1506	0.1073	0.2075
D117_C		0.0416	0.0293	0.0578	0.0452	0.0315	0.0630
D121_C		0.0524	0.0377	0.0747	0.0366	0.0261	0.0516
D150_C		0.0263	0.0193	0.0380	0.0311	0.0239	0.0480
D163_C		0.0192	0.0132	0.0269	0.0228	0.0182	0.0373
D198_C		0.0043	0.0023	0.0065	0.0062	0.0038	0.0096
