# Supplementary material for: Histone Variant HTZ1 Shows Extensive Epistasis with, but Does Not Increase Robustness to, New Mutations
Source: PLoS Genet. 2013 Aug 22;9(8):e1003733. doi: 10.1371/journal.pgen.1003733 (PMC3749942; doi:10.1371/journal.pgen.1003733)
Supplement: Table S4 — Analysis of between-line variance, using PAM instead of PCA for dimensional reduction. P-values are given for Levene's test of differences in between-line variance (column 3), likelihood-ratio test for significance of genotype-by-line interaction term (column 4) and likelihood-ratio test for significance of genotype-by-line interaction term after scaling data to remove spreading (column 7). Estimated percentages of total interaction variance explained by line crossing (column 5) and line spreading (column 6) are also given. * = medoid traits for which HTZ1− lines have a significantly greater between-line variance than HTZ1+ lines (p<0.05 by Levene's test). † = medoid traits for which HTZ1+ lines have a significantly greater between-line variance than HTZ1− lines (p<0.05 by Levene's test). (PDF) [file pgen.1003733.s011.pdf]

Table S4. Analysis of between-line variance, using PAM instead of PCA for dimensional reduction.

Cell Type	Trait	P, Levene's Test	P, Interaction	Crossing	Spreading	Scaled P, Interaction
No Bud	C12.1_A	0.6179	$<10^{-16}$	99.321	0.679	$<10^{-16}$
No Bud	C115_A	0.0043 *	$<10^{-16}$	90.726	9.274	$<10^{-16}$
No Bud	D176_A	0.4284	$<10^{-16}$	99.902	0.098	$<10^{-16}$
No Bud	D16.1_A	0.0004 †	$<10^{-16}$	94.577	5.423	$<10^{-16}$
No Bud	D117_A	0.7772	$<10^{-16}$	99.524	0.476	$<10^{-16}$
No Bud	D148_A	0.0153 *	$<10^{-16}$	93.126	6.874	$<10^{-16}$
Small Bud	C12.1_A1B	0.0001 †	$<10^{-16}$	95.565	4.435	$<10^{-16}$
Small Bud	C107_A1B	0.0743	$<10^{-16}$	97.581	2.419	$<10^{-16}$
Small Bud	C13_A1B	0.0373 *	$<10^{-16}$	95.341	4.659	$<10^{-16}$
Small Bud	C109_A1B	0.0024 *	$<10^{-16}$	69.901	30.099	$<10^{-16}$
Small Bud	D178_A1B	0.1522	$<10^{-16}$	99.306	0.694	$<10^{-16}$
Small Bud	D16.3_A1B	$5.92x10^{-5}$ †	$<10^{-16}$	90.829	9.171	$<10^{-16}$
Small Bud	D104_A1B	0.0636	$<10^{-16}$	97.792	2.208	$<10^{-16}$
Small Bud	D110_A1B	0.3391	$<10^{-16}$	99.363	0.637	$<10^{-16}$
Small Bud	D136_A1B	0.7449	$<10^{-16}$	99.095	0.905	$<10^{-16}$
Small Bud	D170_A1B	0.6101	$<10^{-16}$	97.775	2.225	$<10^{-16}$
Large Bud	C101_C	0.2422	$<10^{-16}$	99.779	0.221	$<10^{-16}$
Large Bud	D166_C	0.2637	0.0218	99.547	0.453	0.0249
Large Bud	D158_C	0.1902	$<10^{-16}$	98.717	1.283	$<10^{-16}$
Large Bud	D185_C	0.0293 †	$<10^{-16}$	97.973	2.027	$<10^{-16}$
Large Bud	C116_C	0.2800	$<10^{-16}$	99.252	0.748	$<10^{-16}$
Large Bud	D103_C	0.2827	$<10^{-16}$	99.657	0.343	$<10^{-16}$
Large Bud	C117_C	0.8700	$<10^{-16}$	99.979	0.021	$<10^{-16}$
Large Bud	D176_C	0.4172	$<10^{-16}$	99.509	0.491	$<10^{-16}$
Large Bud	D177_C	0.0136 *	$<10^{-16}$	98.020	1.980	$<10^{-16}$
Large Bud	D193_C	$3.01x10^{-5}$ †	$<10^{-16}$	90.997	9.003	$<10^{-16}$
Large Bud	D108_C	0.0009 †	$<10^{-16}$	96.482	3.518	$<10^{-16}$
Large Bud	D109_C	0.0051 †	$<10^{-16}$	95.039	4.961	$<10^{-16}$
Large Bud	D117_C	0.7793	$<10^{-16}$	99.841	0.159	$<10^{-16}$
Large Bud	D121_C	0.4272	$<10^{-16}$	99.549	0.451	$<10^{-16}$
Large Bud	D150_C	0.0699	$<10^{-16}$	96.337	3.663	$<10^{-16}$
Large Bud	D163_C	0.3940	$1.7x10^{-14}$	98.900	1.100	$9.91x10^{-14}$
Large Bud	D198_C	0.6301	$<10^{-16}$	99.953	0.047	$<10^{-16}$
