# Supplementary material for: Histone Variant HTZ1 Shows Extensive Epistasis with, but Does Not Increase Robustness to, New Mutations
Source: PLoS Genet. 2013 Aug 22;9(8):e1003733. doi: 10.1371/journal.pgen.1003733 (PMC3749942; doi:10.1371/journal.pgen.1003733)
Supplement: Table S5 — List of MA lines used in this study. Lines that are classified as having a stable-red inheritance pattern are indicated with an X in the stability column (see text). (PDF) [file pgen.1003733.s012.pdf]

Table S5. List of MA lines used in this study.

**Line Name**	**Stability**
MA3	
MA4	
MA9	
MA10	X
MA11	X
MA12	X
MA14	X
MA16	X
MA17	X
MA21	
MA23	
MA24	
MA25	X
MA26	
MA28	
MA31	
MA33	X
MA34	X
MA35	X
MA38	
MA39	X
MA40	
MA42	X
MA44	X
MA50	
MA51	
MA52	
MA57	X
MA58	
MA62	
MA63	
MA65	
MA66	X
MA68	
MA69	
MA72	X
MA73	X
MA74	
MA78	
MA79	X
MA82	
MA84	
MA85	X
MA86	X
MA87	
MA88	
MA89	
MA95	
MA96	X
MA100	X

MA102	X
MA103	
MA104	X
MA106	X
MA110	
MA112	X
MA113	X
MA114	X
MA116	X
MA119	X
MA120	
MA122	
MA123	
MA124	X
MA125	X
MA127	
MA129	
MA131	
MA132	X
MA133	X
MA135	X
MA136	X
MA139	X
MA141	X
MA145	X
MA146	X
MA148	X
MA149	X
MA150	X
