# Supplementary material for: Histone Variant HTZ1 Shows Extensive Epistasis with, but Does Not Increase Robustness to, New Mutations
Source: PLoS Genet. 2013 Aug 22;9(8):e1003733. doi: 10.1371/journal.pgen.1003733 (PMC3749942; doi:10.1371/journal.pgen.1003733)
Supplement: Table S6 — Traits for each cell type measured by CalMorph and used in this study. Each trait is listed by the name used by CalMorph and is accompanied by a brief biological description of what that trait measures. (PDF) [file pgen.1003733.s013.pdf]

Table S6. Traits for each cell type measured by CalMorph and used in this study.

Name	Cell Type	Biological Description
C11.1_A	No Bud	mother area
C12.1_A	No Bud	mother circumference
C13_A	No Bud	elliptical approximation
C103_A	No Bud	long axis length
C104_A	No Bud	short axis length
C115_A	No Bud	axis ratio
C126_A	No Bud	brightness difference of cell wall
D14.1_A	No Bud	nucleus area
D15.1_A	No Bud	nucleus brightness sum
D16.1_A	No Bud	highest brightness nucleus
D17.1_A	No Bud	elliptical approximation of nuclei
D102_A	No Bud	distance between nuclear gravity center and mother tip
D117_A	No Bud	distance between nuclear gravity center and mother center
D127_A	No Bud	distance between nuclear brightest point and nearer cell tip
D135_A	No Bud	distance between nuclear brightest point and mother center
D147_A	No Bud	relative distance of nuclear gravity center in mother to mother center
D148_A	No Bud	relative distance of nuclear brightest point in mother to mother center
D173_A	No Bud	maximal distance between nuclear gravity center and nuclear outline in mother
D176_A	No Bud	nuclear long axis length in mother
D179_A	No Bud	nuclear minimum radius in mother
D182_A	No Bud	nuclear axis ratio in mother
D191_A	No Bud	average nuclear brightness in mother
D194_A	No Bud	maximal intensity of nuclear brightness divided by average in mother
C11.1_A1B	Small Bud	mother area
C11.2_A1B	Small Bud	bud area
C12.1_A1B	Small Bud	mother circumference
C13_A1B	Small Bud	elliptical approximation
C101_A1B	Small Bud	whole cell size (mother and bud)
C102_A1B	Small Bud	whole cell outline length
C103_A1B	Small Bud	long axis length
C104_A1B	Small Bud	short axis length
C105_A1B	Small Bud	neck position
C107_A1B	Small Bud	bud long axis length
C108_A1B	Small Bud	bud short axis length
C109_A1B	Small Bud	neck width
C111_A1B	Small Bud	distance between bud tip and mother long axis extension
C112_A1B	Small Bud	distance between middle point of neck and mother center
C113_A1B	Small Bud	distance between bud tip and mother long axis through middle point of neck.
C114_A1B	Small Bud	bud axis ratio
C115_A1B	Small Bud	mother axis ratio
C116_A1B	Small Bud	axis ratio ratio
C126_A1B	Small Bud	brightness difference of cell wall
C128_A1B	Small Bud	distance between middle point of neck and mother hip.
D14.3_A1B	Small Bud	nucleus area
D15.3_A1B	Small Bud	nucleus brightness sum
D16.3_A1B	Small Bud	highest brightness nucleus
D17.3_A1B	Small Bud	elliptical approximation of nuclei
D104_A1B	Small Bud	distance between nuclear gravity center and mother tip
D107_A1B	Small Bud	ratio of D104_A1B to long axis length
D110_A1B	Small Bud	distance between nuclear gravity center and middle point of neck

D114_A1B	Small Bud	ratio of D110_A1B to C128_A1B
D118_A1B	Small Bud	distance between nuclear gravity center and mother center
D126_A1B	Small Bud	distance between nuclear gravity center and mother hip
D121_C	Large Bud	Distance between nuclear gravity center in bud and bud tip
D123_C	Large Bud	ratio of D121_C to C107_C
D125_C	Large Bud	distance between nuclear gravity center and in mother and mother tip
D128_C	Large Bud	distance between nuclear brightest point in mother and mother tip
D130_C	Large Bud	distance between nuclear brightest point in mother and middle point of neck
D131_C	Large Bud	distance between nuclear brightest point in bud and middle point of neck
D134_C	Large Bud	distance between two nuclear brightest points through middle point of neck
D135_C	Large Bud	distance between nuclear brightest point in mother and mother center
D139_C	Large Bud	distance between nuclear brightest point in bud and bud center
D141_C	Large Bud	distance between nuclear brightest point in mother and mother hip
D143_C	Large Bud	Distance between nuclear outline point and middle point of neck
D144_C	Large Bud	distance between nuclear outline point in bud and middle point of neck
D145_C	Large Bud	Distance between nuclear outline point in mother and mother hip
D146_C	Large Bud	distance between nuclear outline point in bud and bud tip
D147_C	Large Bud	relative distance of nuclear gravity center in mother to mother center
D148_C	Large Bud	relative distance of nuclear brightest point center in mother to mother center
D149_C	Large Bud	relative distance of nuclear gravity center in bud to bud center
D150_C	Large Bud	relative distance of nuclear brightest point in bud to bud center
D151_C	Large Bud	ratio of distance between each nucleus and middle point of neck
D152_C	Large Bud	mobility of nucleus in mother
D153_C	Large Bud	mobility of nucleus in bud
D157_C	Large Bud	angle between lines: short axis to bud nuclear bright point and short axis to bud tip
D158_C	Large Bud	angle between lines: long axis length and line between nuclear center of gravity and nuclear bright spot
D159_C	Large Bud	angle between lines: long axis and line between bud nuclear bright point and bud nuclear center of gravity
D162_C	Large Bud	angle between lines: line between nuclear bright point and nuclear center of gravity and line between neck and long axis.
D163_C	Large Bud	angle between lines: line between bud nuclear bright point and bud nuclear center of gravity and line between neck and long axis.
D166_C	Large Bud	Angle between line: line between nuclear bright point and nuclear center of gravity and line between neck and bud tip
D167_C	Large Bud	angle between lines: line between bud nuclear bright point and bud nuclear center of gravity and line between neck and bud tip
D169_C	Large Bud	angle between lines: neck to nuclear gravity center
D170_C	Large Bud	angle between lines: neck to nuclear bright spot
D173_C	Large Bud	maximal distance between nuclear gravity center and nuclear outline
D174_C	Large Bud	maximal distance between nuclear gravity center and nuclear outline in bud
D176_C	Large Bud	nuclear long axis length in mother
D177_C	Large Bud	nuclear long axis length in bud
D182_C	Large Bud	nuclear axis ratio in mother
D183_C	Large Bud	nuclear axis ratio in bud
D185_C	Large Bud	length from nucleus to neck
D186_C	Large Bud	length from bud nucleus to neck
D191_C	Large Bud	average nuclear brightness in mother
D192_C	Large Bud	average nuclear brightness in bud
D193_C	Large Bud	average nuclear brightness in whole cell
D194_C	Large Bud	maximal intensity of nuclear brightness divided by average in mother
D195_C	Large Bud	maximal intensity of nuclear brightness divided by average in bud
D196_C	Large Bud	maximal intensity of nuclear brightness divided by average in whole cell
D197_C	Large Bud	ratio of nuclear size
D198_C	Large Bud	ratio of nuclear brightness
