# Supplementary material for: Histone Variant HTZ1 Shows Extensive Epistasis with, but Does Not Increase Robustness to, New Mutations
Source: PLoS Genet. 2013 Aug 22;9(8):e1003733. doi: 10.1371/journal.pgen.1003733 (PMC3749942; doi:10.1371/journal.pgen.1003733)
Supplement: Text S1 — Details of model-independent measures of within-line and between-line variance and linear mixed modeling. (PDF) [file pgen.1003733.s014.pdf]

Text S1. Details of model-independent measures of within-line and between-line variance and linear mixed modeling.

Model-independent measures of within-line and between-line variance

Within-line and between-line variances for the HTZ1+ and HTZ1– genotypes were analyzed using using model-independent approaches to confirm the results of estimating variance components using Markov chain Monte Carlo (MCMC) sampling.

The median absolute deviation (MAD) of each trait (principal component) was used to measure within-line variance, as it is less sensitive to non-normality than other measures of spread [67, 68]. However, plotting the raw MADs of each line against the line median tended to show a dependency of the MAD on the median (Figure S2). To correct for this dependency, the residuals of a local (lowess) regression of MAD on median were used in place of raw MADs to compare differences in within-line variation, similar to our previous analysis [41].

For each cell type, the within-line variance of principal components of HTZ1– strains is significantly greater than that of HTZ1+ strains: for unbudded cells: $p = 1.051 \times 10^{-9}$, small-budded cells: $p = 3.686 \times 10^{-8}$, and large-budded cells: $p = 2.602 \times 10^{-9}$ (Wilcoxon signed-rank test). Note that the difference between HTZ1– and HTZ1+ is also significant when using raw MADs, implying that the lowess-based correction is not itself responsible for the difference: unbudded cells: $p = 1.167 \times 10^{-10}$, small-budded cells: $p = 9.296 \times 10^{-10}$, and large-budded cells: $p = 3.782 \times 10^{-10}$.

To test for differences in between-line variance we used Levene's test, as it has been proposed as a robust test for a difference in between-line variance [67]. Each principal component was tested for a difference in between-line variance of HTZ1+ and HTZ1– lines. At a significance threshold of $p < 0.05$, for unbudded cells, two principal components showed a significant difference. For small-budded cells five principal components showed a significant difference, and for large-budded cells, four principal components showed a significant difference (Table S1). Note that this threshold is not corrected for multiple tests, so it is generous to the hypothesis that HTZ1 genotype influences between-line variance. Nonetheless, only 11 of 33 principal components show a significant difference in between-line variance. Of these, the HTZ1+ strains show greater variance in six of the principal components and the HTZ1– strains show greater variance in the other five.

Linear mixed modeling

In addition to using MCMC for variance-component estimation, we fit linear mixed models using restricted maximum likelihood [69]. HTZ1 genotype was modeled as a fixed effect, and MA line and genotype-by-line interaction were modeled as random effects.

Likelihood-ratio tests were used to determine if the data are best explained by a model with or without a line-by-genotype interaction term (for model comparison, models were refit using maximum likelihood and compared using the anova function in R) [69]. For all principal components, the model with the interaction term fit significantly better (Table S1).

The interaction variance from these models can further be partitioned into a term due to line crossing and a term due to line spreading [30, 48, 49], using the equation:

$$V_{g\times l} = \left[sd_{HTZ1+}\, sd_{HTZ1-} \left(1 - \frac{var_L}{var_L + var_{G\times L}} \right) \right] + \left[\frac{\left(sd_{HTZ1+} - sd_{HTZ1-} \right)^2}{2} \right] \quad . \qquad \textbf{(1)}$$

$V_{g\times l}$ is the total genotype-by-line interaction variance. This formula is identical to that given for $V_{g\times l}$ previously [30], except $r_{HTZ1+,HTZ1-}$, a term representing the correlation of line means, is replaced by $var_L / (var_L + var_{G\times L})$ [65]. This change was made because $r_{HTZ1+,HTZ1-}$ had been calculated using mean squares estimates from linear models [30]. This approach is not appropriate for the models used in this study, which are fitted to unbalanced data, so $var_L / (var_L + var_{G\times L})$ was used instead, with var_L and $var_{G\times L}$ derived from the linear models described above. Standard deviations (sd_{HTZ1+} and sd_{HTZ1-}) are derived from linear models fitted to the data separated by genotype, modeling MA line as a random effect. The crossing of line means is represented by the first bracketed term in Equation 1, and the spreading of line means is represented by the second bracketed term in Equation 1. Percentages of the total interaction variance explained by line crossing and spreading are presented in Table S1.

As an additional test of the prevalence of line crossing, the tests for significant genotype-by-line interactions were repeated on data scaled to eliminate differences in the spread of line means [30]. To standardize the data, the difference between each value and its genotype mean was divided by the standard deviation of line means grouped by genotype (the between-line standard deviation). Figure S4 shows the standardized data, which illustrate the extent of line crossing that remains. Linear models with interaction terms fit these standardized data better than models without the interaction term (Table S1). This analysis further supports the presence of a significant genotype-by-line interaction that is due to the crossing of line means and not differences in between-line variance. Very similar results were obtained when using medoids rather than principal components (Table S4).

References

67. Dworkin I. (2005) Canalization, cryptic variation, and developmental buffering: A critical examination and analytical perspective. In: Hallgrimsson B, Hall BK, editors. Variation: A Central Concept in Biology. San Diego: Elsevier Academic Press. pp. 131-158.

68. Schultz BB. (1985) Levene's test for relative variation. Syst Biol 34: 449-456.

69. Pinheiro J, Bates D. (2000) Mixed-effects models in S and S-PLUS. Berlin: Springer.
